# Genome-wide analysis of fitness data and its application to improve metabolic models

**DOI:** 10.1186/s12859-018-2341-9

**Published:** 2018-10-10

**Authors:** Edward Vitkin, Oz Solomon, Sharon Sultan, Zohar Yakhini

**Affiliations:** 1 Department of Computer Science, Technion, Haifa, Israel; 2 Faculty of Biotechnology and Food Engineering, Technion, Haifa, Israel; 3 School of Computer Science, The Interdisciplinary Center, Herzliya, Israel

**Keywords:** Fitness data, Metabolic modelling, Orphan reactions, Co-fitness, Co-expression, Flux balance analysis (FBA)

## Abstract

**Background:**

Synthetic biology and related techniques enable genome scale high-throughput investigation of the effect on organism fitness of different gene knock-downs/outs and of other modifications of genomic sequence.

**Results:**

We develop statistical and computational pipelines and frameworks for analyzing high throughput fitness data over a genome scale set of sequence variants. Analyzing data from a high-throughput knock-down/knock-out bacterial study, we investigate differences and determinants of the effect on fitness in different conditions. Comparing fitness vectors of genes, across tens of conditions, we observe that fitness consequences strongly depend on genomic location and more weakly depend on gene sequence similarity and on functional relationships. In analyzing promoter sequences, we identified motifs associated with conditions studied in bacterial media such as Casaminos, D-glucose, Sucrose, and other sugars and amino-acid sources.

We also use fitness data to infer genes associated with orphan metabolic reactions in the iJO1366 *E. coli* metabolic model. To do this, we developed a new computational method that integrates gene fitness and gene expression profiles within a given reaction network neighborhood to associate this reaction with a set of genes that potentially encode the catalyzing proteins. We then apply this approach to predict candidate genes for 107 orphan reactions in iJO1366. Furthermore - we validate our methodology with known reactions using a leave-one-out approach. Specifically, using top-20 candidates selected based on combined fitness and expression datasets, we correctly reconstruct 39.7% of the reactions, as compared to 33% based on fitness and to 26% based on expression separately, and to 4.02% as a random baseline. Our model improvement results include a novel association of a gene to an orphan cytosine nucleosidation reaction.

**Conclusion:**

Our pipeline for metabolic modeling shows a clear benefit of using fitness data for predicting genes of orphan reactions. Along with the analysis pipelines we developed, it can be used to analyze similar high-throughput data.

**Electronic supplementary material:**

The online version of this article (10.1186/s12859-018-2341-9) contains supplementary material, which is available to authorized users.

## Background

Progress in sequencing techniques has greatly improved our understanding of bacterial genomes [1, 2]. In parallel, technologies that support modifying the genomic sequences of living organisms, including bacteria [3–5], enable targeting of known loci in the genome. The combination of these developments facilitates studying of bacterial gene function by physically modifying related sequences in living genomes and measuring the phenotypic effects triggered by such modifications. An important example of this emerging technique is organism fitness profiles [5, 6], where organism growth rates in different conditions and under different genomic modifications are measured. Progress in the quality and scope of synthetic DNA libraries and in applying them to studying regulation in living cells [7–10], as well as more affordable sequencing methods, support higher throughput approaches to phenotypic analysis of synthetically modified genomes.

In this study, we develop statistical and computational pipelines and analysis techniques that are useful in the context of analyzing high-throughput fitness data over a genome scale set of sequence variants. We demonstrate the use of the approach and the pipelines developed by analyzing TnSeq *E. coli* data from Wetmore et al. [5]. In TnSeq, long interfering sequences are inserted in recoverable positions of the genome [5, 6]. We analyze the differences, in terms of fitness effects, between insertions in different functional parts of the genome: promoter regions, coding sequences (CDS) and un-translated regions (UTR). Analyzing fitness data and promoter sequences, we find promoter enriched motifs for 88% of the conditions. For example, this approach yields two enriched motifs that are associated with amino-acid biosynthesis. We also analyze the correlation of the insertions resulted effects (co-fitness) that modify related regions, gene paralogs, genes which are in close proximity in the genome, similar protein domains, and genes on the same operon. On the phenotype level, we compare the observed co-fitness to co-expression, inferred from expression profiling studies [11, 12], and interestingly find only very minimal agreement.

The understanding of bacterial genomes enables the use of metabolic models for designing bacterial production systems and other synthetic biology devices. Genome-scale metabolic network models leverage the existing knowledge of organism biochemistry and genetics to construct a framework for simulating processes. The core of the metabolic model is the information about the stoichiometry of the metabolic reactions and the associations between protein coding genes, and the reactions that they catalyze [13]. iJO1366 [14], which is the latest model of *Escherichia coli K-12 MG1655,* contains information about 1366 genes, 1136 unique metabolites and 2251 metabolic reactions, out of which 128 reactions are orphan (70 metabolic and 58 transport), meaning that they are not associated with any gene.

An important part of the methodology developed in this paper is the use of high throughput fitness data to infer genes that potentially encode for proteins catalyzing orphan reactions. Current approaches rely on the idea that genes and reactions in the local neighborhood have similar behavioral profiles. The exact definition of these profiles is deduced from the nature of the available biological data, such as sequence similarity (phylogenetic profile), sequence genomic context, gene-metabolome associations, gene expression data and others. For the best of our knowledge, none of the recent metabolic modeling studies proposes a method to improve the assignment of genes to reactions using fitness assays alone or incorporated with additional data sources [15–21].

The proposed mathematical framework is developed and tested over the iJO1366 *E. coli* model. We report top-20 predicted candidate genes for each orphan reaction and further substantiate some of the findings based on existing literature. For example – we identify a gene that codes to a cytosine nucleosidation reaction (CMPN), that is an orphan reaction in the current model.

In summary, the contribution of this paper consists of:

- A methodology and a pipeline for analyzing high-throughput bacterial fitness data, including specific statistical approaches.
- Novel analysis of non-coding insertions in TnSeq data.
- A new framework for improving metabolic models based on high-throughput fitness data only, as well as in combination with expression data.
- Freely available software implementation of some of the methods is provided along with this manuscript (see Additional file 3).
- Biological findings, including motifs associated with the tested conditions, characterization of the relationship between co-expression and co-fitness, and genes that potentially encode proteins that catalyze *E. coli* orphan reactions.

## Results

### An analysis pipeline for high throughput fitness data, including metabolic model improvement

**Fig. 1:**
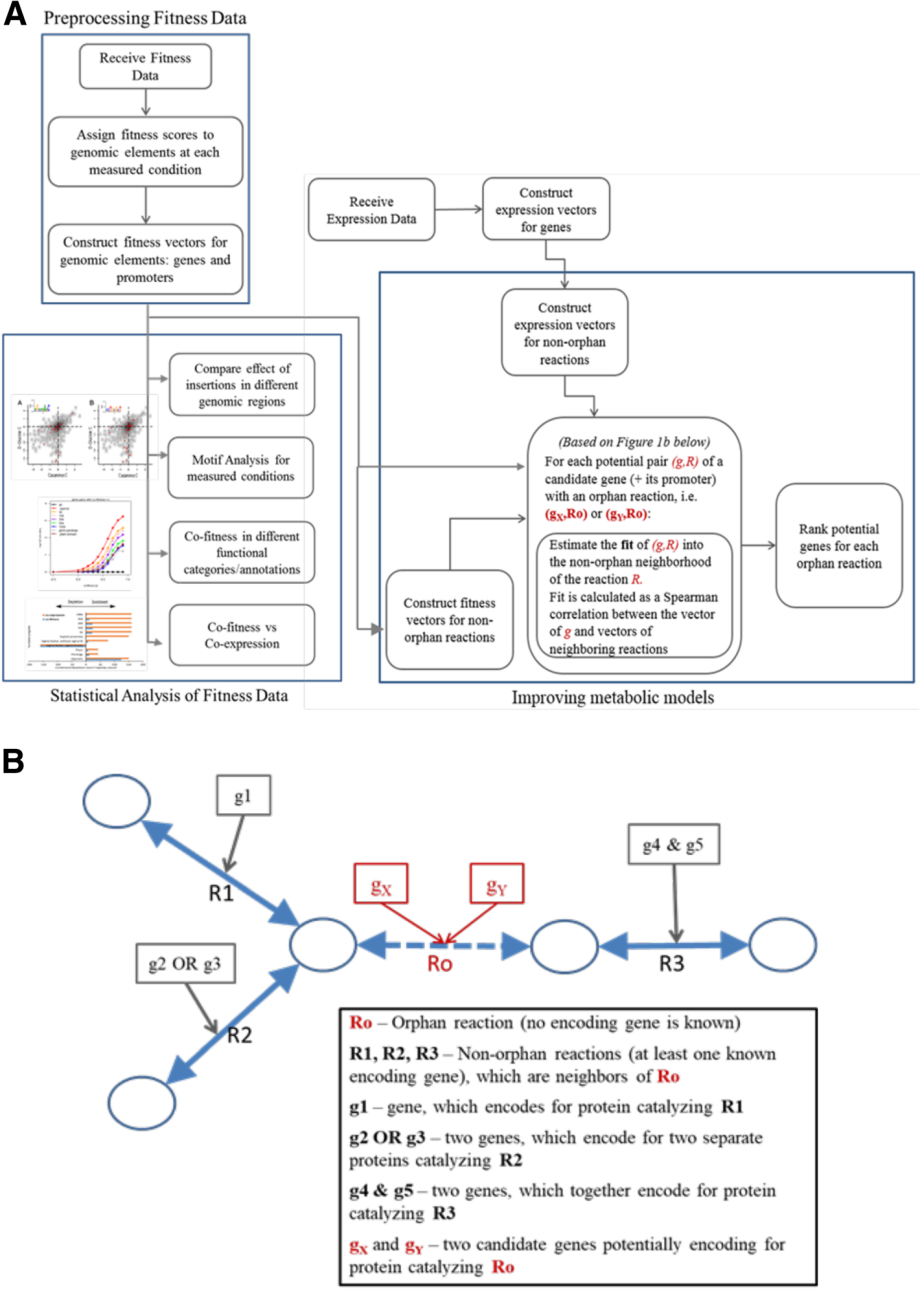
Analyses and methods used in the current study. **a** The general workflow. **b** example of orphan and non-orphan reactions

In the current study we present statistical analysis methods for fitness data to explore bacterial gene regulation and to improve metabolic modeling. The complete pipeline we developed is outlined in Fig. 1a (with further details in Methods). In brief, we first incorporate fitness data such as, for example, data from Wetmore et al. [5]^1^ and assign fitness scores for any genomic element under investigation (including non-coding regions that were not analyzed in the original publication). We construct fitness vectors, across conditions, for genes and their promoters. At the end of this stage we have a matrix of genes and/or genomic locations, across conditions, with fitness scores as entries. We use the fitness vectors to compare the effects of insertions in different genomic regions, search for common motifs in promoter regions and compare fitness profiles to gene transcription profiles. Finally, we use the same fitness vectors to improve metabolic models and to predict genes that regulate orphan reactions (Fig. 1b), as explained in detail in the Methods.

### Genome-wide analysis of fitness data – Genomic and functional context

#### Assessment of fitness effects for different gene parts

We characterized the effect of insertions in different gene parts by comparing the distributions of fitness measurements in each of the investigated conditions of Wetmore et al. [5] (Methods). Namely, we investigated whether insertions in coding regions, UTRs and promoters have different overall effect magnitudes. To this aim, we used the raw strain fitness scores as reported within. We did so using raw supplementary data without the further normalization steps reported in Wetmore et al., and considered distributions obtained for promoters, UTRs and coding regions. A heatmap representation of the results (Additional file 1: Figure S1A) shows that different regions have distributions of fitness scores centered around different averages. Interestingly, it seems that UTRs have more negative scores than CDS in most of the conditions (Additional file 1: Figure S1A).

Indeed, when testing 3’UTRs, we found that in 30 out of 48 conditions (62.5%), the insertions in 3’UTRs have stronger, average negative fitness effect compared to the insertions in other gene parts (Additional file 1: Figure S1A). Under a uniform null model this observation has a *p*-value of 4.41 × 10^− 8^ (tail of *Binom(48,0.25)* at 30, as we considered here 4 types of regions: promoter, CDS and 5’UTR and 3’UTR). However, when using percentiles, 10%, 25% and 50% (median) of the fitness values, the *p*-values were not significant (binomial test *p*-value > 0.25).

When examining the low 10% of the fitness values (Additional file 1: Figure S1B), representing insertions with the greatest effect on fitness, we see stronger effect of promoter regions in 23 out of 48 conditions (47.9%). Under a uniform null model this observation has a *p*-value of 4.9 × 10^− 4^ (tail of *Binom(48,0.25)* at 23).

In stratifying promoters according to the regulation of sigma factors, we found that in 36 out of 48 conditions (75%) insertions in *sigma28* dependent promoters have stronger negative average fitness effect than insertions in other promoters. *Sigma28* is responsible for the initiation of transcription of genes related to motility and flagella synthesis [22]. Under a uniform null model this has a *p*-value of 4.37 × 10^− 21^ (tail of *Binom(48,0.143)* at 36, as we considered 7 types of promoters). When using percentiles, 10%, 25% and 50% (median) of the fitness values, we found a similar trend with 16 out of 48, 23 out of 48, and 28 out of 48, respectively (binomial test *p*-value =0.0007, 2.89 × 10^− 8^, and 1.88 × 10^− 12^, respectively under the *Binom*(48,0.143) null).

#### Promoter motif analysis

High-throughput fitness data can be useful in the context of discovering or understanding regulatory sequence motifs. To further asses motifs related to fitness in the measured conditions, promoter regions of *E. coli* (genome assembly: NC_000913.2) were intersected with insertions from Wetmore et al. [5]. To infer fitness effect of insertions in promoters these were further analyzed as described in Methods and in Additional file 1: Figure S2.

In 994 of out of 1128 (88.1%) pairs of conditions we found at least one enriched PSSM with corrected mmHG *p*-value< 0.01 using DRIMust [23] (Methods). Motifs with strong statistical significance hypothetically represent binding sites that are used by factors involved in growth under the analyzed conditions as exemplified below.

**Fig. 2:**
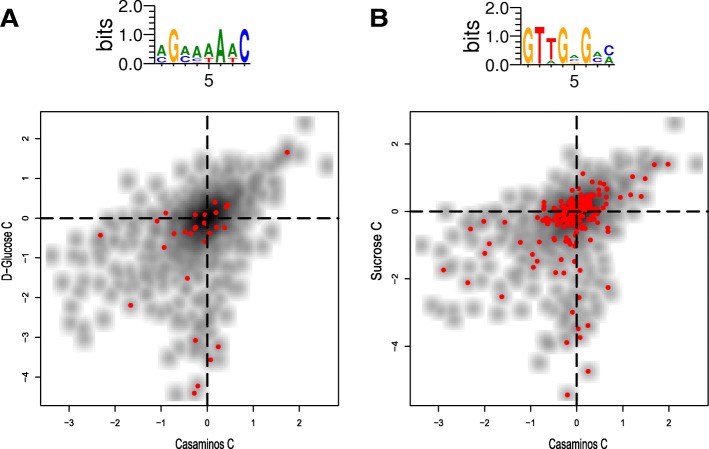
**a** First enriched motif in D-Glucose C. vs. Casaminos C. (the second enriched motif is found in Figure S3A).**b** Enriched motif detected for Sucrose C vs. Casaminos C. A comparison of this motif to the known metJ motif is found in Additional file 1: Figure S3B. Red points are promoters with high PSSM values with respect to the given motif. Corrected mmHG p-value <0.01 for both panels. Shaded black points are all the promoters analyzed

Figure 2 depicts two examples. Figure 2a depicts a motif enriched in D-Glucose C vs. Casaminos C. Each point is a promoter; in red – all promoters with sufficiently high PSSM values with respect to the given motif. The corrected mmHG *p*-values are 0.0042 and 0.0094, for Fig. 2a and Additional file 1: Figure S3A, respectively (Methods). We can see that a relatively high number of red points, representing the presence of the motifs, are aligned to the x = 0 line where there is no effect in Casaminos, but for many cases a strong effect in D-Glucose C. Analyzing Sucrose C vs. Casaminos C (Fig. 2b), we observed a corrected mmHG *p*-value = 9.61 × 10^− 5^ and a motif which is similar to metJ (methionine repressor) binding site (according to both Tomtom [24] and Stamp [25], Additional file 1: Figure S3B), a repressor of Met biosynthesis [26]. This result points to the importance of the regulation of Met biosynthesis under Sucrose, and to the fact that it is likely regulated by metJ binding to its transcription factor binding site (TFBS). Interestingly, the two lowest (with respect to y-axis) red points in Fig. 2b are from uncharacterized promoters that reside in ilvC (b3774) and serA (b2913) coding sequence. Both ilvC and serA have correlated fitness values (Spearman *R* = 0.66, *p*-value = 3.38 × 10^− 7^) and regulate amino acid synthesis.

#### Analysis of co-fitness

**Fig. 3:**
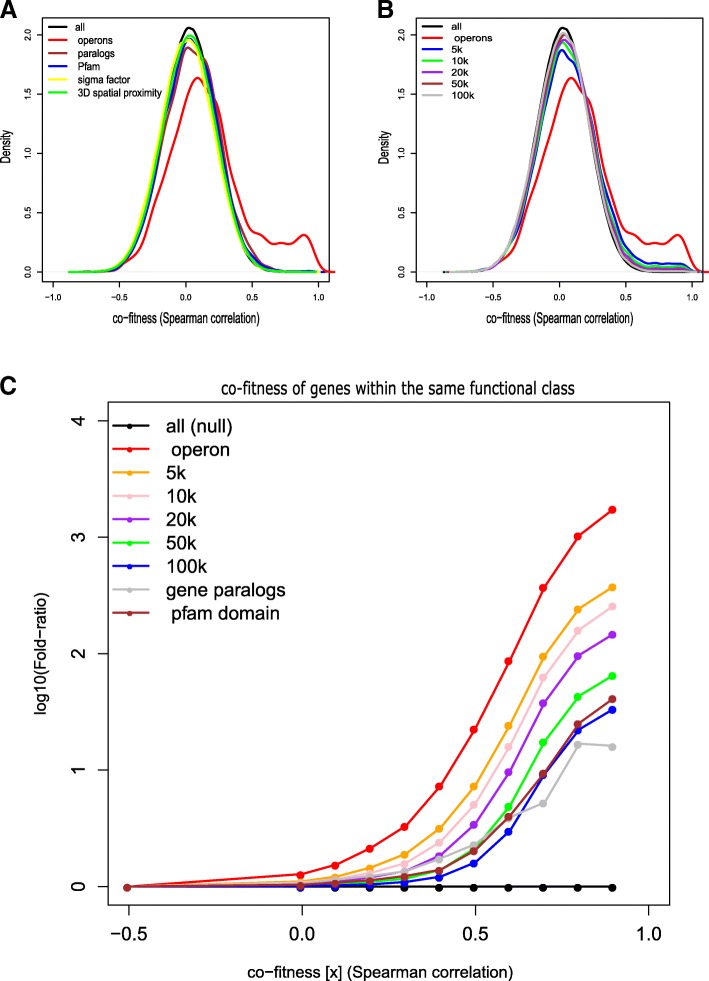
The distributions of co-fitness using different functional classess to group gene pairs. **a** Different classes from annotation databases (Methods). **b** Grouping the gene pairs according to genomic position bins. **c** The fraction of gene pairs with co-fitness ≥x divided by the correponding fraction of gene pairs in the background distribtion (total gene pairs, null). For further details see Methods

We calculated the gene-gene fitness Spearman correlation, hereinafter co-fitness, to look for similarities in fitness profiles, across all conditions, in different functional classes (Methods). We compared the distributions of gene pair co-fitness values in each of standard class to the total co-fitness distribution (Fig. 3a). We found that operon co-fitness is significantly high relative to the total co-fitness (one-tail Wilcoxon *p*-value = 2.7 × 10^− 124^). Gene paralogs and genes with similar functional domains (at least one common domain, as in Pfam DB) also have significantly higher co-fitness values (one-tail Wilcoxon *p*-values of 10^− 12^ and 1.4 × 10^− 8^, respectively).

**Fig. 4:**
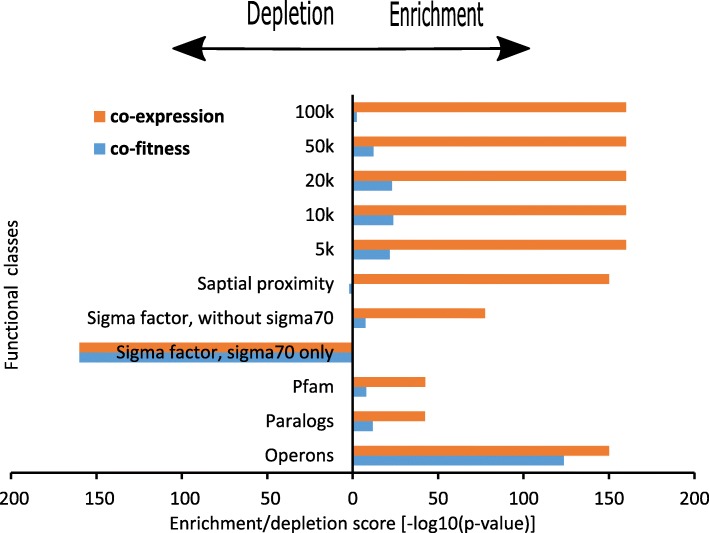
Enrichment/depletion scores (one tail Wilcoxon, -log10(p-value)) for annotated gene pairs grouped according to functional classes. Orange: co-expression. Blue: co-fitness

Interestingly, grouping genes according to their genome position results in high co-fitness even after removing gene pairs located within the same operon. This signal becomes weaker as the distance increases, however stays significant even in long distances (Fig. 3b, one-tail Wilcoxon *p*-value < 10^− 20^ for 5 kb, 10 kb and 20 kb bins; for 50 kb *p*-value< 10^− 12^ and for 100 kb *p*-value = 0.004). This finding may suggest that even distant regulatory regions influence the gene functionality. This trend could also reflect the functional clustering of nearby genes that extends beyond operons [27]. Interestingly, gene pairs that reside close in the genome show higher co-fitness than genes with similar sequence or genes that share similar functional domains (Fig. 3c and Fig. 4).

In order to avoid GC content bias that may result with high co-fitness for proximal genes with similar GC content (Wetmore et al. [5]), we also restricted our analysis to genes pairs with different GC content (Methods). Indeed, after using this additional filter, the co-fitness of proximal genes that are not in the same operon is still found to be enriched, with distances of 5 kb, 10 kb, 20 kb and 50 kb, yielding Wilcoxon test *p*-value< 10^− 12^ and for 100 kb, Wilcoxon test *p*-value = 0.003 (only in 200 kb the result is not significant anymore).

Annotating the gene pairs according to similar Sigma factors or genes that reside in 3D spatial proximity (data from Xie et al. [28]) showed no statistically significant increase in co-fitness signal as compared to the total co-fitness distribution – the null model (note, in contrast to gene co-expression as described below).

#### Comparison of co-fitness and co-expression profiles

The results described in the gene-reaction assignment section (Section “Gene-to-Reaction assignment”) show an advantage for using fitness over expression in the context of metabolic model improvement. In this section, we compare the characteristics of gene co-expression and gene co-fitness in *E. coli*. Using gene expression data from [11] (GEO id: GSE32561) we calculated co-expression for gene pairs. We used the same functional classes as above to explore co-expression profiles and for comparing co-expression profiles with co-fitness profiles.

When comparing co-fitness and co-expression in the context of the functional classes used above, co-expression yields more significantly enriched results (lower *p*-values, when compared to its background co-expression distribution. See Fig. 4 and Additional file 1: Figure S4). Interestingly, gene pairs that are spatially proximal (Xie et al. [28]) show higher than expected co-expression, while their co-fitness was not significantly different from the background co-fitness distribution. To investigate co-fitness and co-expression within targets of different Sigma factors, we considered gene pairs that are regulated by *sigma70* and gene pairs that are regulated by other Sigma factors. We found that gene pairs regulated by *sigma70* show lower co-fitness and co-expression than the total gene pairs (Fig. 4). We observe enrichment for gene pairs aggregated by the rest of the sigma factors where higher co-fitness and co-expression scores were observed (Fig. 4). This result reflects the fact that most of *E. coli* genes are regulated by *sigma70*, therefore show close to background correlations. On the other hand, specific Sigma factors like *sigma54* [29] are related to specific pathways in the bacteria and therefore their regulated genes are correlated.

Common function and other functional associated properties can be affected in (and therefore possibly inferred from) co-fitness as well as in co-expression - similar gene expression patterns across conditions. In a more detailed representation of co-expression against co-fitness, none of the classes showed strong signal (Additional file 1: Figure S5). Examples for two pairs of genes that are correlated in their fitness but not in their expression, or vice versa, are available in Additional file 1: Figure S6. As shown in Table 1, we observed very low correlation between co-expression and co-fitness (all Spearman R values are < 0.2). This trend is in agreement with previous fitness studies [6, 30]. However, assessing the mmHG scores for some of the analyzed classes [31, 32], we see that the top parts of both lists (of ranked co-expression and co-fitness pairs) have significant overlap (Table 1). In conclusion, co-expression and co-fitness are minimally correlated, mostly in the high values, and may add information to each other.

**Table 1:** Correlation and mmHG results for comparing co-fitness to co-expression. Empirical *p*-values for the mmHG tests were calculated based on shuffled data, where each list, used as functional class for the gene pairs, was shuffled (100 instances), preserving the original partition structure

	Spearman’s R	Corrected mmHG statistics	# of pairs (N)	B	n*	b*	Empirical *p*-value from shuffled data
All pairs	0.007	0.1	6,485,401	43	368	2	N/A
Same operons	0.186	1.18 × 10^−13^	2453	251	966	165	0
Paralog genes	0.048	1	4584	5	46	2	0.32
Same Pfam domain	0.051	1	10,626	948	155	31	0.47
Within 5 kb	0.058	2.53 × 10^−4^	7443	221	991	61	0
Within 10 kb	0.046	2.49 × 10^−4^	14,202	57	481	14	0

### Gene-to-reaction assignment

High throughput fitness data can act as a basis for validating, iterating and improving genome-scale metabolic networks. We developed a novel approach for utilizing fitness data to predict genes associated to orphan reactions, which we then tested on iJO1366 metabolic model of *E. coli* [14] using fitness data from Wetmore et al. [5] (Methods). To the best of our knowledge there are no recent metabolic modeling studies that used fitness data for this purpose. In this section, we demonstrate the potential of leveraging fitness data alone as well as integrated gene expression profiles. Also, we present some of our novel findings that map genes to *E. coli* orphan reactions.

#### Performance evaluation based on non-orphan reaction data

**Fig. 5:**
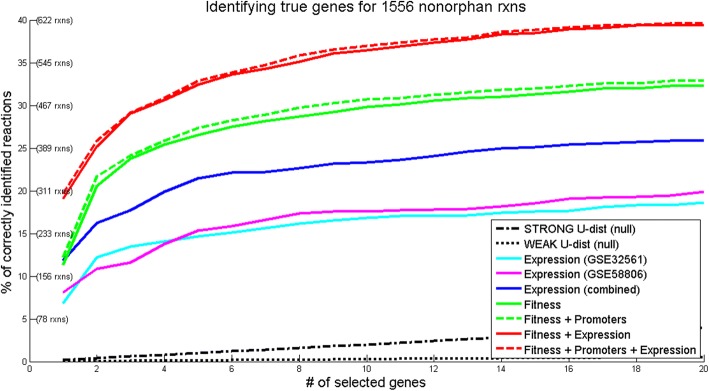
Validation. Comparative accuracy of Association Likelihood Score (ALS, Methods) values. For every k (on the x-axis) we indicate the fraction of reactions for which the true gene is within the predicted top k. ALS computed based on Spearman correlation to the two best neighbors

To assess the quality of assigning genes to reactions using our approach (Methods) we cast non-orphan reactions as orphans, one reaction at a time, and test our ability to reconstruct the known hidden assignment of at least one original gene. For each such reaction, we calculate the rank of the true known gene or genes out of all candidate genes. Figure 5 and Additional file 1: Table S1 depict the performance of this validation test for predicting a reaction gene for 1556 non-orphan reactions.

Additional file 1: Table S1 clearly shows that all the scoring approaches perform significantly better than random reaction assignments. More importantly, assignments based on fitness data were significantly better than those based on expression data – 503 vs 404 (24.5% improvement) correctly identified gene-reaction pairs in top-20 candidates. Considering both types of data produced even better results of 614 correct predictions in top-20 candidates for 1556 validation reactions (52% increase). Finally, incorporation of promoter data boosts the performance on average by 0.5–2%. The combined prediction accuracy based on fitness data together with promoter and expression data is 39.7%, when considering the top-20 candidates, and 19.7% accuracy at predicting at the top candidate, which is respectively 52.7% and 66.3% better than accuracy based solely on expression data. Analysis of accuracy for the prediction of a second reaction gene for 565 validation reactions with at least two genes is further addressed in Figure S12 and Additional file 1: Table S2.

#### Predicting genes for orphan reactions

Assignment of genes to orphan reactions was performed using the combined fitness and expression scores with incorporation of promoter fitness data. We identified 107 (Methods) adequate orphan reactions and assigned them genes (the prediction results can be found in Additional file 2).

We verified the predictions with EcoCyc data [33] and further substantiated some of our findings based on this cross comparison. For example, for the orphan transporter reaction ALAt2rpp (L-alanine reversible transport via proton symport cytoplasm-periplasm) the first predicted candidate (with Bayesian confidence of ~ 4% and unbiased confidence of ~ 98.97%, see Methods) is cycA (b4208), which can act as an L-alanine transporter [34, 35]. Another example is the internal reaction CMPN (CMP nucleosidase: “*CMP + H2O→Cytosine + αD-Ribose-5P*”), for which the first predicted candidate (with Bayesian confidence of 23% and unbiased confidence of ~ 99.85%), is rihC (b0030), which is known as “ribonucleoside hydrolase 3” catalyzing, among others, the “*Cytidine+H2O→D-ribofuranose + Cytosine*” reaction [36]. This knowledge is not captured in the model and is revealed by our analysis.

## Discussion

In this study, we describe novel approaches and computational pipelines to analyzing high-throughput fitness data and their application in improving metabolic modeling. Certain biological insights have been gained along with the pipelines explained above. Interestingly, non-coding regions were shown to be more sensitive to TnSeq insertions than coding regions, in contrast to the common belief that CDS insertions carry the most significant consequences. Indeed, some important regulators of gene expression bind to non-coding regions and modification of these sequences would interrupt their binding. A caveat on this statement relates to annotation inaccuracy, what looks like a non-coding part can be an unknown coding part of some gene.

Comparing co-fitness to co-expression using different functional classes, we found that some classes lead to more coordinated expression than to coordinated growth differences (fitness). Interestingly, gene pairs that reside in spatial proximity [28] have higher co-expression than expected at random. This trend was not observed for co-fitness. This finding may be due to the fact that transcription is coordinated in space, while fitness, which is affected by more regulatory steps, is more difficult to spatially coordinate. Xie et al. [28] already suggested the existence of transcription factories in bacteria and these were also pointed out in other organisms [37–41]. For other classes, we also observe co-expression to be more enriched than co-fitness (comparing correlations within the class to total background. See Fig. 4). Again – the higher complexity of co-fitness as well as factors related to the measurement may explain these differences. Deutschbauer et al. [6] have previously shown very minimal agreement between differential gene expression and fitness differences. The identified differences/commonalities may also be influenced by the technical heterogeneity of the data used. Using non-parametric statistics, we can avoid some of these biases (Additional file 1: Figure S5 and Table 1). Still, additional work is needed to better assess this link.

**Fig. 6:**
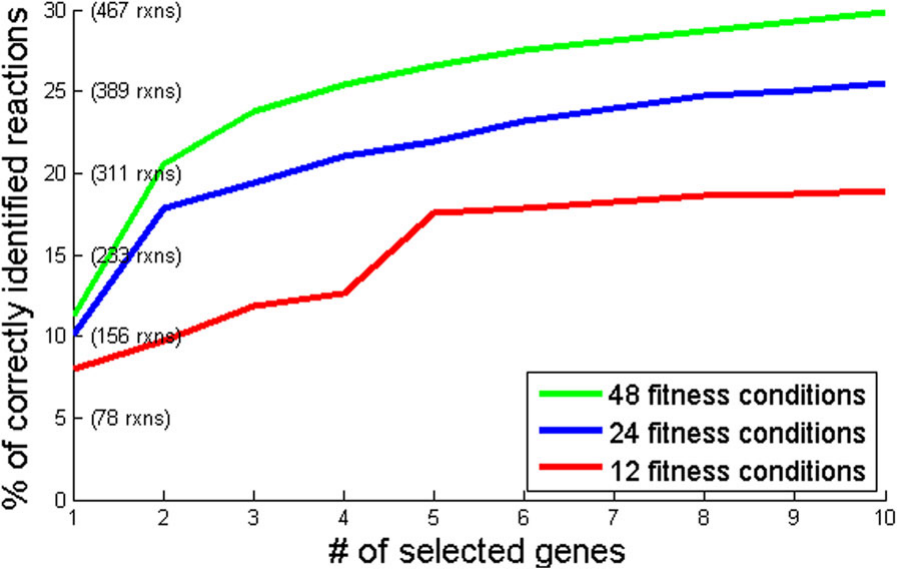
Prediction performance depends on the number of conditions measured

Our statistical and computational approaches provide pipelines for the first data analysis steps toward understanding and interpreting data from fitness screens. We also take a step further in the context of metabolic modelling. High throughput fitness data can act as a basis for validating, iterating and improving models by directly comparing results to predictions in knock-down and knock-out approaches. We directly demonstrate one potential utility - the prediction of genes that encode for proteins catalyzing model orphan reactions. In this context, we show the value of knock-down or knock-out high-throughput fitness studies. Indeed, incorporating fitness data into our metabolic modeling was shown here to clearly improve over the random baselines. We also analyze the dependence of the performance of this approach on the number of conditions measured, and observe a clear benefit gained by increasing the number of measured conditions (Fig. 6). Moreover, we demonstrate that integration of fitness data with expression data significantly outperforms the usage of each dataset separately. The latter suggests that incorporation of additional types of data, such as protein-protein interaction (PPI) or phylogeny, might further improve the prediction results for metabolic modeling.

## Conclusions

Our proposed methodology, both for genome-wide analysis of fitness data and for its use in the context of metabolic model inference, sets the stage to developing more computational methods and tools and to applications in new datasets [42]. The availability of fitness screens in organisms or systems that are less well characterized, combined with the use of methods presented here, can drive model development for these contexts.

## Methods

The current study and the methods we used are outlined in Fig. 1.

### Obtaining the fitness data

In the current study, we used Wetmore et al. [5] data as a comprehensive dataset for fitness in *E. coli* in 48 different media conditions. We analyzed both coding and non-coding regions. For coding sequence regions (CDS) we used fitness scores directly reported in Wetmore et al (http://genomics.lbl.gov/supplemental/rbarseq/html/Keio/fit_logratios_good.tab). For non-coding insertions (insertions in non-coding regions of the genome, i.e. promoters and un-translated regions, UTRs), which were not analyzed in the original paper, we calculated average fitness scores as explained below. Annotations of non-coding regions in *E. coli* were taken from RegulonDB and EcoCyc [33, 43]. We denote an insertion in a genomic region position *p* by *Ins(p)*. Considering a non-coding region *nc* and media condition *γ*, we compute:

1 $$ fitness\_ score\ \left( nc,\gamma \right)=\frac{1}{\mid \pi\ (nc)\mid}\sum \limits_{\pi\ (nc)} fitness\_ score\left( Ins(p),\gamma \right) $$

Where *π* (*nc*) = {*Ins*(*p*) : *p* ∈ *nc*}, i.e. all insertions in genomic positions within the considered non-coding region. In other words, all the fitness scores related to genomic positions within a non-coding region are averaged in order to score this region. The value *fitness* _ *score*(*Ins*(*p*), *γ*) is taken from the raw insertion results table provided by Wetmore et al.

While comparing between non-coding and coding fitness values (as in Additional file 1: Figure S1 and related Results section), we used for both coding and non-coding the same normalization scheme, namely averaging the scores (as in Eq. 1). This in order to not bias this analysis, as in the original paper by Wetmore et al. [5], where they further normalized the coding fitness values by using a weighted average of strain fitness values and removing lowly abundant samples.

For each genomic element of interest (either coding or non-coding) we define its *fitness vector* as a vector combining its *fitness_score* over all available conditions γ in the dataset.

### Promoters motif analysis

For each condition γ and for all promoters, we rank the promoter sequences*, seq(prom),* where *prom* is a given promoter*,* according to the values of *fitness_score(prom, γ)*. The ranked sequence lists were input into DRIMust [23, 44], setting detection of motif to double stranded DNA, with minimum and maximum motif lengths 6 and 20, respectively. The output PSSMs were further assessed using the mmHG statistics [31, 32]. Similar steps, summarized in Additional file 1: Figure S2, were also used for analyzing pairs of conditions. Here for each pair of conditions *γl*, *γk* we compute:

2 $$ fitness\_ score\ \left( prom,\gamma l,\gamma k\right)= fitness\_ score\ \left( prom,\gamma l\right)- fitness\_ score\ \left( prom,\gamma k\right) $$

Perl and Java code that automate motif analysis on multiple conditions can be found in the supplementary information of this manuscript (Additional file 3).

To find similarity of the found motifs to known binding sites in bacteria, we used both Tomtom [24] and Stamp [25] webservers with their default parameters (organism: *E. coli*, database: dpinteract).

### Co-fitness and co-expression

We define the *co-fitness* of two genomic elements (genes, promoters, etc.) as Spearman correlation of corresponding fitness vectors. Similarly, the *co-expression* is defined as Spearman correlation of corresponding expression vectors.

To investigate co-fitness profiles and their relationship to other properties of gene regulation, functional classes of *E. coli* were collected from different sources. Operons information was collected from RegulonDB and EcoCyc [33, 43]. The Cluster of Orthologous Groups of proteins (COG) [45] was used as the source for *E. coli* gene paralogs. Annotations for genes located in spatial proximity in the *E. coli* genome (3D space) were taken from Xie et al. [28], considering the 75th percentile as the proximity threshold. Information regarding operons and the sigma factors regulating them was taken from RegulonDB [43]. We also culled the *E. coli* genes according to common Pfam domains (from Pfam DB [46]). In addition, we used 5 kb, 10 kb, 20 kb, 50 kb and 100 kb bin sizes to group the genes according to their genomic position.

To compute relative co-fitness for different classes as in Fig. 3c we proceeded as follows: For − 0.5 ≤ x ≤ 1 and for a class of pairs C, we compute:

$\rho \left(x,C\right)=\frac{\# of\ pairs\ in\ C\  with\  CF\ge X}{\# of\ pairs\ in\ C}$, where CF is the co-fitness value.

We also compute: $\kern1.25em \rho \left(x,T\right)=\frac{Total\# of\ pairs\kern0.5em with\  CF\ge X}{Total\# of\ pairs}$, where T is the total gene-gene pairs in the analysis. The log_10_ fold ratio for C and x is: log_10_$\frac{\rho \left(x,C\right)}{\rho \left(x,T\right).}$

The same classes also guided our comparison between co-fitness and co-expression profiles (as in Fig. 4). As the signal from operons is relatively strong (Results) we removed same operon genes pairs from the analyses of the other functional classes.

To avoid biases that related to similar GC content of proximal genes (while analyzing co-fitness of proximal genes that are not part of the same operon), we calculated genes CDS GC content from the *E. coli* genome (NC_000913.2) using GeneBank *E. coli* gene annotation. According to the genes’ GC content, we divided them into GC content bins. Gene pairs that both genes are included in the same bin (same GC content 10% percentile) were filtered out.

#### Metabolic models

Commonly used computational approaches for predicting biological organism behavior using metabolic models are based on Flux Balance Analysis (FBA). FBA analyzes internal reaction fluxes under the assumption that the modelled organism metabolic network is regulated to maximize some cellular objective under a predefined set of constraints. FBA-based approaches have a wide range of applications including phenotype analysis, bioengineering and metabolic model reconstruction [13, 20, 47–52].

The reaction stoichiometry in a metabolic model is represented by stoichiometric matrix *S*, wherein *S*_*m,r*_ corresponds to stoichiometric coefficient of metabolite *m* in the reaction *r*. The vector of metabolic fluxes that are carried by the model reactions, normally denoted as $\overrightarrow{v}$, is constrained both by mass-balance (Eq. 3a) and by maximal/minimal feasible fluxes *v*^*UB*^ and *v*^*LB*^ (Eq. 3b). In addition, the addressed analysis question may impose additional context-specific constraints [20, 51, 52].

3a $$ S\cdot \overrightarrow{v}=0 $$

3b $$ {v}_r^{LB}\le {v}_r\le {v}_r^{UB}\kern2em :\kern1.5em \forall r\in Reactions $$

Reactions in the model are called *orphan reactions* if no corresponding catalyzing gene is known for them. Other reactions are called *non-orphan*. Each non-orphan reaction *r*_*i*_ may be associated with one or several genes (up to 17 in the iJO1366 [14] model of *E.coli* as shown in Additional file 1: Figure S7). Each such gene *g* is responsible (alone or by cooperation with other genes) for coding and/or activating a protein (enzyme) that catalyzes *r*_*i*_. Mathematically, we represent a set of genes working together to construct a protein by an Λ (AND) clause of binary indicators representing gene activity (existence, functionality, expression). If certain reaction can be activated by one of several alternative proteins, then the activity status of this reaction is represented herein by an V (OR) clause of binary indicators of activities of each protein (Eq. 4a). For example, the regular expression for the reaction *rx* catalyzed either by protein *p1*, encoded by single gene *g1*, or by protein *p2*, encoded by a combination of genes *g2* and *g3*, is represented in (Eq. 4b).

4a $$ activity\left({r}_i\right)=\underset{p(i)=1}{\overset{\# Proteins\left({r}_i\right)}{\mathrm{V}}}\left\{\underset{g_j^{p(i)}=1}{\overset{\# Genes\left(p(i)\right)}{\Lambda}}\left[{g}_j^{p(i)}\right]\right\}\kern1.5em :\kern1em {g}_j^{p(i)}=\left\{\begin{array}{l}1\kern0.5em -\kern0.5em iff\kern0.5em {\mathrm{g}}_{\mathrm{j}}^{p\left(\mathrm{i}\right)}\kern0.5em is\kern0.5em active\\ {}0\kern0.5em -\kern0.5em otherwise\end{array}\right. $$

4b $$ activity(rx)= activity\left({p}_1\right)\ \mathrm{V}\  activity\left({p}_2\right)=\left({g}_1\right)\ \mathrm{V}\ \left({g}_2\Lambda {g}_3\right) $$

When a gene gets knocked out for a given strain, such as in fitness screens, then the corresponding term in the above reaction regular expression becomes *FALSE*.

### Fitness vectors for non-orphan reactions

A major challenge in constructing metabolic models is an assignment of genes to orphan reactions. The majority of automatic approaches addressing this issue [20, 53–55] operate under the reasonable assumption that the behavior of each reaction (both orphan and non-orphan) is highly correlated with the behavior of the reaction neighborhood (Section “Reaction neighborhood”). Here, we also use this logic while addressing the reaction behavior in the context of the gene fitness data. To do so we need a definition of the fitness of non-orphan reactions based on the fitness measurements of its genes. We define the organism fitness after the knockout of a certain reaction to be the maximum over the fitness values of the disjunctive clauses, which, in turn, are the minimum over individual gene fitness values under the conjunction sign (Eq. 5a). To continue the specific example described above in the context of Eq. 4b, the fitness of *rx* in the condition γ is described in (Eq. 5b).

5a $$ fitness\_ score\left({r}_i,\gamma \right)=\underset{p(i)=1}{\overset{\# Proteins\left({r}_i\right)}{\max }}\left\{\underset{g_j^{p(i)}=1}{\overset{\# Genes\left(p(i)\right)}{\min }}\left\{ fitness\_ score\left({g}_j^{p(i)},\gamma \right)\right\}\right\} $$

5b $$ fitness\_ score\left( rx,\gamma \right)=\max \left\{\begin{array}{l} fitness\_ score\left({g}_1,\gamma \right)\kern1em \\ {}\min \left\{\begin{array}{l} fitness\_ score\left({g}_2,\gamma \right)\\ {} fitness\_ score\left({g}_3,\gamma \right)\end{array}\right\}\end{array}\right\} $$

Once created, reaction fitness values are Z-normalized per each condition as in Eq. 6, where *RXN* denotes the set of all considered reactions and *ZFS(r*_*i*_*, γ)* denotes Z-normalized fitness score of reaction *r*_*i*_ in condition *γ*.

6 $$ ZFS\left({r}_i,\gamma \right)=\frac{fitness\_ score\left({r}_i,\gamma \right)-\underset{r\in RXN}{MEAN}\left\{ fitness\_ score\left(r,\gamma \right)\right\}}{\underset{r\in RXN}{STD}\left\{ fitness\_ score\left(r,\gamma \right)\right\}} $$

A fitness vector for a given reaction *r*_*i*_, denoted as *ZFV(r*_*i*_*)*, is now constructed using *ZFS(r*_*i*_*, γ)*, for all conditions γ in the dataset.

### Fitness vectors for genes and promoters

A fitness vector for a gene *g* is directly taken from all measurements reported for *g* in Wetmore et al. [5], running across all conditions *γ* in the dataset and using Z-normalized values (similar to Eq. 6). We denote this vector by *ZFV(g).*

Assignment of genes to orphan reactions may also benefit from the information available for the gene promoters. Clearly, if a candidate gene for some orphan reaction is highly correlated with the reaction neighborhood, but its known promoter does not support such correlation – this information should be incorporated in the total ranking of this gene with respect to other candidates. Thus, we adapt the Eqs. 1–6 to construct *ZFV(p),* the vector of Z-normalized fitness scores for the promotor *p*, running across all conditions.

### Reaction neighborhood

Metabolic reactions connected to each other by shared metabolites are called *neighboring reactions*. Such *neighboring reactions* may have very similar activity patterns, i.e. there is a high chance that a reaction is active when its neighbor is active. Such activity similarities are most evident on linear metabolic pathways, when products of one reaction are transferred as input substrates to another. However, that’s not the case for all shared metabolites. Metabolites like H2O, H+ and others appear in extremely high numbers of reactions, thus taking them into account for neighborhood calculations is almost always noisy. We call them *high-frequency* metabolites.

To correctly assess the neighborhood of a given reaction *r*, we greedily (from high to low frequencies) remove from its equation all high-frequency (frequency higher than 11) metabolites unless such removal leads to less than two non-orphan neighbors remaining for *r* or for some other reaction (Additional file 1: Figure S8). The constraint of two non-orphan neighbors is required to support linear fluxes. The high-frequency cutoff 11 was selected according to the distribution of metabolite frequencies in iJO1366 *E. coli* model (Additional file 1: Figure S9).

We define a reaction to be adequate for neighborhood analysis (in short – *adequate*) if after deletion process as above, it has two or more non-orphan neighbors with assigned fitness vectors. Out of 1787 non-orphan reactions we found 1556 to be adequate, which were used for validation purposes (Results). Out of 128 orphan reactions we found 107 to be adequate, meaning that we can propose candidate genes for 83.6% of orphan reactions.

### Fitness vectors for orphan reactions. Gene-to-reaction assignment based on reaction neighborhoods

For the orphan reactions, i.e. reactions without any known associated gene, the method described above (Section “Fitness vectors for non-orphan reactions”) for construction of fitness vectors is obviously not appropriate. However, we can *guess* that certain candidate gene *candG* is an encoding gene for the given orphan reaction *Ro*. If this guess is correct, it is reasonable to expect high similarity of the fitness behavior of *candG* with the fitness behavior of the genes on the non-orphan reactions in its neighborhood. We measure this similarity using a Spearman correlation test. For each adequate orphan reaction *Ro* we define the *Association Likelihood Score* (*ALS*) of assigning the candidate gene *candG* to *Ro* as the mean Spearman correlation of the fitness vector of *candG* to the 2 most-correlated fitness vectors of non-orphan neighbors of *Ro* (Eq. 7). The number “2” was selected as the minimal value, which allows solving single orphan reaction gap in a linear pathway.

7 $$ ALS\left( Ro, candG\right)=\frac{1}{2}\left(\begin{array}{l} Spearman\left[ ZFV(candG), ZFV\left({neighb}_1(Ro)\right)\right]\\ {}\kern15em +\\ {} Spearman\left[ ZFV(candG), ZFV\left({neighb}_2(Ro)\right)\right]\end{array}\right) $$

Here *neighb*_*1*_*(Ro)* and *neigb*_*2*_*(Ro)* are the two neighbor reactions of *Ro* with fitness vectors most correlated to *ZFV*(*candG*) (Spearman correlation). Once each candidate gene is scored, the genes are ranked, and the top 20 candidates are reported (Additional file 2).

The performance of this method was evaluated using leave-one-out approach with non-orphan reactions where the goal was to predict at least one of the known reaction genes (Results).

### Promoter-to-reaction assignment based on reaction neighborhoods

The process of assignment of promoters to reactions is very similar to the assignment of genes. First, for each non-orphan reaction we construct its fitness vector as described in Section “Fitness vectors for non-orphan reactions”. Then, we compute the Spearman correlation between this vector and the *ZFV(candP)* for all candidate promoters. Finally, for an adequate orphan reaction *Ro,* we compute the maximal average Spearman correlation *ZFV(candP)* attains with pairs of non-orphan neighbors (similar to Eq. 6).

### Combination of gene and promoter fitness

Gene-to-reaction Spearman-based assignment scores can be adjusted according to promoter-to-reaction Spearman-based association. Indeed, this is valid for genes with existing mapping to promoters, as acquired from RegulonDB and EcoCyc [33, 43]. We define the adjusted (gene, promoter)-to-reaction assignment score as follows (Eq. 8):

8 $$ ALS\left( Ro, candG+\left\{\begin{array}{l}{candP}_1\\ {}{candP}_2\\ {}\dots \end{array}\right\}\right)= ALS\left( Ro, candG\right)+\alpha \sum \limits_i ALS\left( Ro,{candP}_i\right) $$

Where {*candPi*} is the set of all the promoters associated with *candG* or an empty set if there is no such information about promoters exists. We have tested different values of α and the best performance was obtained at α = 0.21.

### Uniform gene-to-reaction assignment null models

Two baselines strawman gene-to-reaction assignment scores were used to represent uniform assignment of genes to reactions as a basis for comparison.

First, under *WEAK U-dist*, we assume that random selection of top *G* genes for *R* reactions will successfully identify $$ \raisebox{1ex}{${R}^{\ast }G$}\!\left/ \!\raisebox{-1ex}{$ TotalGenes$}\right. $$ of true reaction genes. For example, random selection of top 10 out of 3646 candidate genes for 1556 non-orphan reactions will identify true genes for $$ \raisebox{1ex}{${10}^{\ast }1556$}\!\left/ \!\raisebox{-1ex}{$3646$}\right.\approx 4.3 $$reactions.

Second, under *STRONG U-dist*, we assume that the predicted genes are sampled from the set of true reaction genes only (i.e. each sampled gene is a true encoding gene for some non-orphan reaction). Moreover, since some reactions are activated by several genes, at each step average number of genes per reaction will be identified. That is, selection of top *G* genes will identify G*Avg(genes per rxn) of true reaction genes. For example, selection of top 10 candidate genes with average of 3.126 true genes per reaction in iJO1366 model will identify true genes for 10*3.126 ≈ 31.26 reactions.

### Reaction expression vectors and score calculation

We also compare the methodology of the current study with the more standard approach that uses co-expression as a basis for gene to reaction assignment [20, 55].

Two gene expression datasets were used to associate reactions to expression vectors. The first is GSE32561 from Goh et al., which includes 11 gene expression microarray measurements [11]. The second is GSE58806 from Keating et al., which includes 36 gene expression measurements [12]. As a preprocessing step, we omitted expression data for genes which were not covered in Wetmore and colleagues’ fitness analysis [5]. Additional file 1: Figure S10 explains the known model genes covered by the fitness and expression data.

Expression values were first Z-normalized per each condition separately (in the spirit of Eq. 6). Second, reaction expression vectors were constructed in a manner similar to that used for reaction fitness vectors (in the spirit of Eq. 5a). Third, we computed the Spearman correlation between this vector and the expression vectors of every candidate gene. Finally, for an adequate orphan reaction *Ro* we estimated the average Spearman correlation of each candidate gene to two most-correlated non-orphan neighbors (in the spirit of Eq. 7). The combined expression score was defined as average of scores for each dataset separately, that performed better than a score based on vector concatenation.

### Combination of fitness and expression-based scores

The combined score based on fitness and expression data was defined as average between the score resulted from each data source separately:

9 $$ {ALS}_{comb}\left( Ro, candG\right)=\raisebox{1ex}{$1$}\!\left/ \!\raisebox{-1ex}{$2$}\right.{ALS}_{fit}\left( Ro, candG\right)+\raisebox{1ex}{$1$}\!\left/ \!\raisebox{-1ex}{$2$}\right.{ALS}_{expr}\left( Ro, candG\right) $$

### Confidence of gene to reaction assignment

We define confidence of each gene-to-reaction assignment as probability of receiving an assignment score *σ* for true gene-reaction pair in both Bayesian (Eq. 10a) and unbiased (Eq. 10b) approach. The distribution of scores obtained for spurious pairs is compared to that obtained for known pairs in Additional file 1: Figure S11.

10a $$ {\displaystyle \begin{array}{c} Bayesian\\ {} confidence\end{array}}\left( ALS\left(g,r\right)=\sigma \right)=\frac{P\left(\left\{ ALS\left(g,r\right)\ge \sigma \right\}|\left\{g\in genes(r)\right\}\right)\times P\left(g\in genes(r)\right)}{P\left( ALS\left(g,r\right)\ge \sigma \right)} $$

10b $$ {\displaystyle \begin{array}{c} Unbiased\\ {} confidence\end{array}}\left( ALS\left(g,r\right)=\sigma \right)=\frac{P\left(\left\{ ALS\left(g,r\right)\ge \sigma \right\}|\left\{g\in genes(r)\right\}\right)}{P\left(\left\{ ALS\left(g,r\right)\ge \sigma \right\}|\left\{g\in genes(r)\right\}\right)+P\left( ALS\left(g,r\right)\ge \sigma |\left\{g\notin genes(r)\right\}\right)} $$

Where *P*({*g* ∈ *genes*(*r*)}) ≈ 0.044% is the general probability of correct gene-reaction assignment, as calculated on the set of non-orphan reactions and the genes measured by Wetmore et al. [5].

## Additional files

Additional file 1: A word file, includes the supplementary figures (**Figures S1-S12**) and supplementary tables (**Tables S1** and **S2**). (DOCX 980 kb)

Additional file 2: Genes prediction for orphan reactions. In a separate excel file, which can also be downloaded from: http://wassist.cs.technion.ac.il/~edwardv/fitnessAnalysis/Sup2.Genes_prediction_for_orphan_reactions.xlsx. (XLSX 533 kb)

Additional file 3: Perl and Java code for the automation of promoter motif analysis according to fitness scores. Can also be downloaded from: http://wassist.cs.technion.ac.il/~edwardv/fitnessAnalysis/Sup3.motif_analysis_and_fitness_scripts.tar.gz. (GZ 763 kb)

## Abbreviations

ALS: Association likelihood score
CDS: Coding sequence
CMPN: Cytosine nucleosidation reaction
COG: Cluster of orthologs
DB: Database
FBA: Flux balance analysis
mmHG: Minimum-minimum-Hyper-Geometric
PPI: Protein-protein interaction
UTR: Untranslated region

## Footnotes

1
http://genomics.lbl.gov/supplemental/rbarseq/html/Keio/strain_fit.tab

## References

[1] Riley M, Abe T, Arnaud MB, Berlyn MKB, Blattner FR, Chaudhuri RR (2006). Escherichia coli K-12: A cooperatively developed annotation snapshot - 2005. Nucleic Acids Res [Internet].

[2] Zhou J, Rudd KE (2013). EcoGene 3.0. Nucleic Acids Res [Internet]. Oxford University Press.

[3] Van Opijnen T, Camilli A (2013). Transposon insertion sequencing: A new tool for systems-level analysis of microorganisms. Nat Rev Microbiol [Internet]. Nature Research.

[4] Bikard D, Euler CW, Jiang W, Nussenzweig PM, Goldberg GW, Duportet X (2014). Exploiting CRISPR-cas nucleases to produce sequence-specific antimicrobials. Nat Biotechnol [Internet]. Nature Research.

[5] Wetmore KM, Price MN, Waters RJ, Lamson JS, He J, Hoover CA (2015). Rapid quantification of mutant fitness in diverse bacteria by sequencing randomly bar-coded transposons. MBio [Internet]. American Society for Microbiology.

[6] Deutschbauer A, Price MN, Wetmore KM, Shao W, Baumohl JK, Xu Z, et al. Evidence-based annotation of gene function in Shewanella oneidensis MR-1 using genome-wide fitness profiling across 121 conditions. Richardson PM, editor. PLoS Genet [Internet]. Public Library of Science; 2011;7:e1002385. [cited 2017 Oct 2] Available from: http://dx.plos.org/10.1371/journal.pgen.1002385 10.1371/journal.pgen.1002385 PMC3219624 22125499

[7] Sharon E, Van Dijk D, Kalma Y, Keren L, Manor O, Yakhini Z (2014). Probing the effect of promoters on noise in gene expression using thousands of designed sequences. Genome Res [Internet]. Cold Spring Harbor Laboratory Press.

[8] Weingarten-Gabbay Shira, Elias-Kirma Shani, Nir Ronit, Gritsenko Alexey A., Stern-Ginossar Noam, Yakhini Zohar, Weinberger Adina, Segal Eran (2016). Systematic discovery of cap-independent translation sequences in human and viral genomes. Science.

[9] Melnikov A, Murugan A, Zhang X, Tesileanu T, Wang L, Rogov P (2012). Systematic dissection and optimization of inducible enhancers in human cells using a massively parallel reporter assay. Nat Biotechnol [Internet].

[10] Sharon E, Kalma Y, Sharp A, Raveh-Sadka T, Levo M, Zeevi D (2012). Inferring gene regulatory logic from high-throughput measurements of thousands of systematically designed promoters. Nat Biotechnol [Internet].

[11] Goh EB, Baidoo EEK, Keasling JD, Beller HR (2012). Engineering of bacterial methyl ketone synthesis for biofuels. Appl Environ Microbiol.

[12] Keating DH, Zhang Y, Ong IM, McIlwain S, Morales EH, Grass JA (2014). Aromatic inhibitors derived from ammonia-pretreated lignocellulose hinder bacterial ethanologenesis by activating regulatory circuits controlling inhibitor efflux and detoxification. Front Microbiol.

[13] Edwards JS, Covert M, Palsson B (2002). Metabolic modelling of microbes: the flux-balance approach. Environ Microbiol Blackwell Science Ltd.

[14] Orth JD, Conrad TM, Na J, Lerman JA, Nam H, Feist AM (2011). A comprehensive genome-scale reconstruction of *Escherichia coli* metabolism-2011. Mol Syst Biol EMBO and Macmillan Publishers Limited.

[15] Chae TU, Choi SY, Kim JW, Ko Y-S, Lee SY (2017). Recent advances in systems metabolic engineering tools and strategies. Curr Opin Biotechnol [Internet]. Elsevier Current Trends.

[16] Saha Rajib, Chowdhury Anupam, Maranas Costas D (2014). Recent advances in the reconstruction of metabolic models and integration of omics data. Current Opinion in Biotechnology.

[17] Rai A, Saito K (2016). Omics data input for metabolic modeling [Internet]. Curr Opin Biotechnol Elsevier Current Trends.

[18] Chen L, Vitkup D (2006). Predicting genes for orphan metabolic activities using phylogenetic profiles. Genome Biol BioMed Central.

[19] Green ML, Karp PD (2007). Using genome-context data to identify specific types of functional associations in pathway/genome databases. Bioinformatics Oxford University Press.

[20] Vitkin E, Shlomi T (2012). MIRAGE: a functional genomics-based approach for metabolic network model reconstruction and its application to cyanobacteria networks. Genome Biol.

[21] Fuhrer T, Zampieri M, Sévin DC, Sauer U, Zamboni N (2017). Genomewide landscape of gene–metabolome associations in *Escherichia coli*. Mol Syst Biol Wiley-Blackwell.

[22] Arnosti DN, Chamberlin MJ (1989). Secondary sigma factor controls transcription of flagellar and chemotaxis genes in Escherichia coli. Proc Natl Acad Sci U. S. A. [Internet].

[23] Leibovich L, Paz I, Yakhini Z, Mandel-Gutfreund Y (2013). DRIMust: a web server for discovering rank imbalanced motifs using suffix trees. Nucleic Acids Res [Internet]. Oxford University Press.

[24] Gupta S, Stamatoyannopoulos JA, Bailey TL, Noble W, Maniatis T, Goodbourn S (2007). Quantifying similarity between motifs. Genome Biol BioMed Central.

[25] Mahony S, Benos PV (2007). STAMP: A web tool for exploring DNA-binding motif similarities. Nucleic Acids Res [Internet]. Oxford University Press.

[26] Ghochikyan A, Karaivanova IM, Lecocq M, Vusio P, Arnaud MC, Snapyan M (2002). Arginine operator binding by heterologous and chimeric ArgR repressors from Escherichia coli and Bacillus stearothermophilus. J Bacteriol.

[27] Korbel JO, Jensen LJ, Von Mering C, Bork P (2004). Analysis of genomic context: Prediction of functional associations from conserved bidirectionally transcribed gene pairs. Nat Biotechnol [Internet]. Nature Publishing Group.

[28] Xie T, Fu L-Y, Yang Q-Y, Xiong H, Xu H, Ma B-G (2015). Spatial features for *Escherichia coli* genome organization. BMC Genomics [Internet]. BioMed Central.

[29] Francke C, Groot Kormelink T, Hagemeijer Y, Overmars L, Sluijter V, Moezelaar R (2011). Comparative analyses imply that the enigmatic sigma factor 54 is a central controller of the bacterial exterior. BMC Genomics [Internet].

[30] Gelperin DM, White MA, Wilkinson ML, Kon Y, Kung LA, Wise KJ (2005). Biochemical and genetic analysis of the yeast proteome with a movable ORF collection. Genes Dev [Internet]. BioMed Central.

[31] Leibovich L, Yakhini Z (2014). Mutual enrichment in ranked lists and the statistical assessment of position weight matrix motifs. Algorithms Mol Biol BioMed Central.

[32] Steinfeld I, Navon R, Ach R, Yakhini Z (2013). MiRNA target enrichment analysis reveals directly active miRNAs in health and disease. Nucleic Acids Res [Internet].

[33] Keseler IM, Mackie A, Santos-Zavaleta A, Billington R, Bonavides-Martínez C, Caspi R (2017). The EcoCyc database: reflecting new knowledge about Escherichia coli K-12. Nucleic Acids Res Oxford University Press.

[34] Schneider F, Krämer R, Burkovski A (2004). Identification and characterization of the main β-alanine uptake system in Escherichia coli. Appl Microbiol Biotechnol Springer-Verlag.

[35] Robbins JC, Oxender DL (1973). Transport systems for alanine, serine, and glycine in Escherichia coli K 12. J Bacteriol.

[36] Petersen C, Møller LB (2001). The RihA, RihB, and RihC ribonucleoside hydrolases of Escherichia coli. Substrate specificity, gene expression, and regulation. J Biol Chem.

[37] Ay F, Bailey TL, Noble WS (2014). Statistical confidence estimation for Hi-C data reveals regulatory chromatin contacts. Genome Res [Internet]. Cold Spring Harbor Laboratory Press.

[38] Ben-Elazar S, Chor B, Yakhini Z (2016). Extending partial haplotypes to full genome haplotypes using chromosome conformation capture data. Bioinformatics [internet]. Oxford University press.

[39] Dekker J, Marti-Renom MA, Mirny LA (2013). Exploring the three-dimensional organization of genomes: interpreting chromatin interaction data [internet]. Nat Rev Genet NIH Public Access.

[40] Weng X, Xiao J, Elowitz MB, Losick R, Shapiro L (2014). Spatial organization of transcription in bacterial cells. Trends Genet Elsevier.

[41] Duan Z, Andronescu M, Schutz K, McIlwain S, Kim YJ, Lee C (2010). A three-dimensional model of the yeast genome. Nature Nature Research.

[42] Price MN, Wetmore KM, Waters RJ, Callaghan M, Ray J, Liu H (2018). Mutant phenotypes for thousands of bacterial genes of unknown function. Nature [Internet].

[43] Gama-Castro S, Salgado H, Santos-Zavaleta A, Ledezma-Tejeida D, Muñiz-Rascado L, García-Sotelo JS (2016). RegulonDB version 9.0: High-level integration of gene regulation, coexpression, motif clustering and beyond. Nucleic Acids Res [Internet].

[44] Eden E, Lipson D, Yogev S, Yakhini Z (2007). Discovering motifs in ranked lists of DNA sequences. PLoS Comput Biol Public Library of Science.

[45] Tatusov RL (2000). The COG database: a tool for genome-scale analysis of protein functions and evolution. Nucleic Acids Res [Internet]. Oxford University Press.

[46] Finn RD, Coggill P, Eberhardt RY, Eddy SR, Mistry J, Mitchell AL (2016). The Pfam protein families database: Towards a more sustainable future. Nucleic Acids Res [Internet]. Oxford University Press.

[47] Jiang R, Linzon Y, Vitkin E, Yakhini Z, Chudnovsky A, Golberg A (2016). Thermochemical hydrolysis of macroalgae Ulva for biorefinery: Taguchi robust design method. Sci Rep Nature Publishing Group.

[48] Stelling J, Klamt S, Bettenbrock K, Schuster S, Gilles ED, Stelling J (2002). Metabolic network structure determines key aspects of functionality and regulation. Nature.

[49] Bordbar A, Monk JM, King ZA, Palsson BO (2014). Constraint-based models predict metabolic and associated cellular functions. Nat Rev Genet.

[50] Orth Jeffrey D, Thiele Ines, Palsson Bernhard Ø (2010). What is flux balance analysis?. Nature Biotechnology.

[51] Burgard AP, Pharkya P, Maranas CD (2003). OptKnock: a Bilevel programming framework for identifying gene knockout strategies for microbial strain optimization. Biotechnol Bioeng.

[52] Tepper N, Shlomi T (2009). Predicting metabolic engineering knockout strategies for chemical production: accounting for competing pathways. Bioinformatics.

[53] Green ML, Karp PD (2004). A Bayesian method for identifying missing enzymes in predicted metabolic pathway databases. BMC Bioinformatics.

[54] Kharchenko P, Chen L, Freund Y, Vitkup D, Church GM (2006). Identifying metabolic enzymes with multiple types of association evidence. BMC Bioinformatics.

[55] Kharchenko P, Vitkup D, Church GM (2004). Filling gaps in a metabolic network using expression information. Bioinformatics.

