# Supplementary material for: Genome-wide analysis of fitness data and its application to improve metabolic models
Source: BMC Bioinformatics. 2018 Oct 10;19:368. doi: 10.1186/s12859-018-2341-9 (PMC6180484; doi:10.1186/s12859-018-2341-9)
Supplement: Supplementary file 1 — A word file, includes the supplementary figures (Figures S1-S12) and supplementary tables (Tables S1 and S2). (DOCX 980 kb) [file 12859_2018_2341_MOESM1_ESM.docx]

**Supplementary figures**


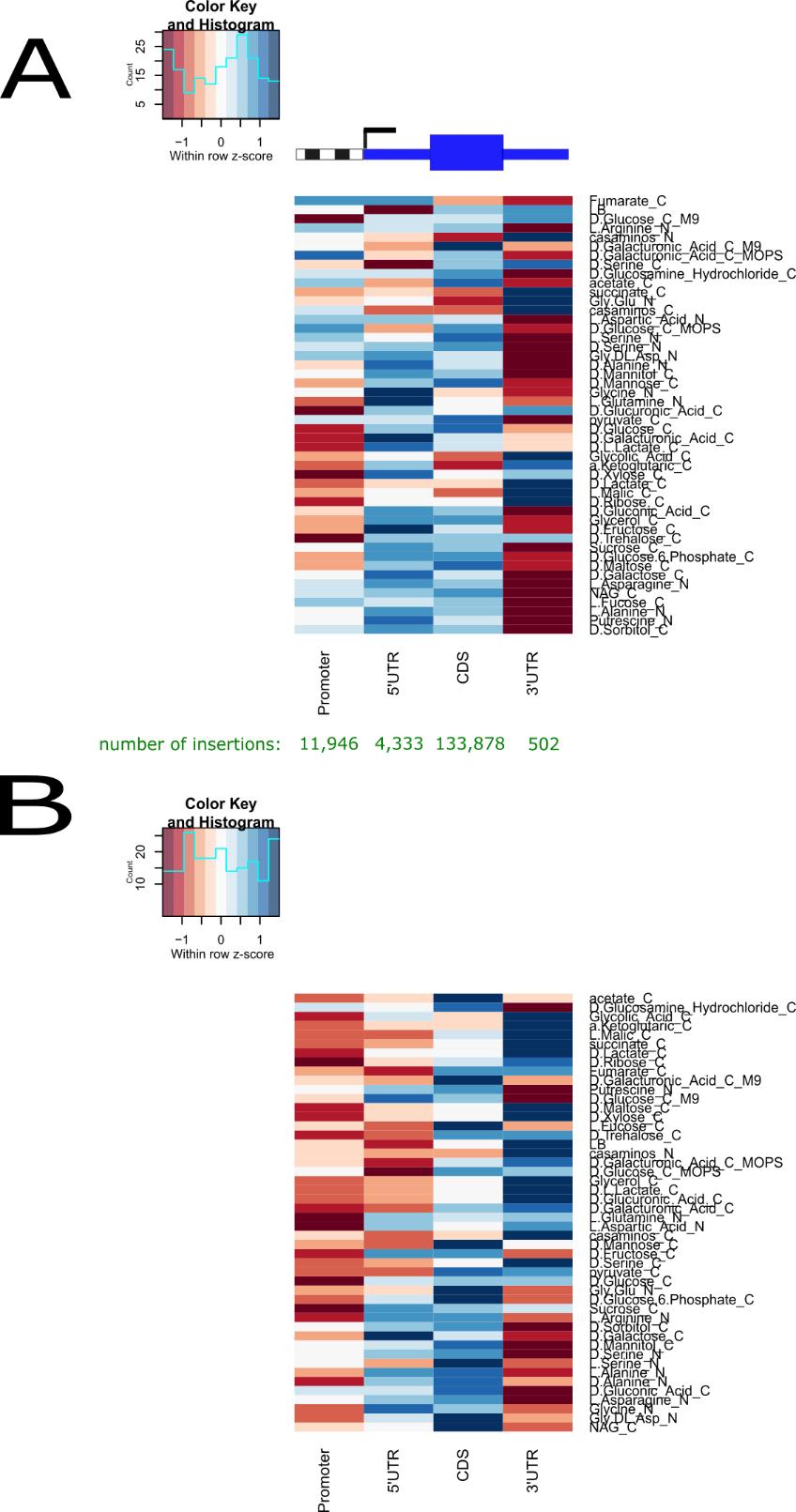


**Fig. S1. A.** Averaged fitness scores across different gene parts. **B.** The low 10% of the fitness scores for each gene part. Gene parts considered are: promoter, 5’UTR, CDS, 3’UTR. In both A and B, the conditions (rows) were transformed into z-scores. Number of insertions in each genomic region is describe below the figure (colored in green). See Methods for detailed explanation about the fitness scores calculation.


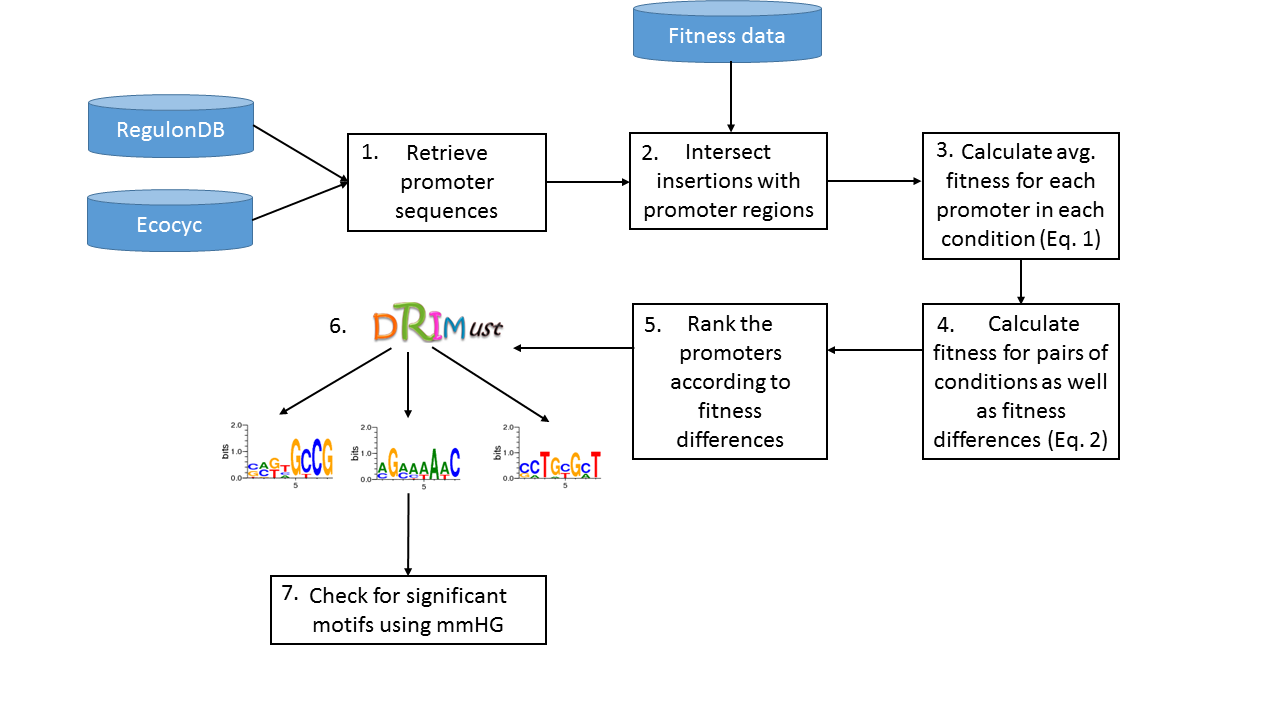


**Fig. S2.** Pipeline for identifying promoter motifs using ranked fitness data.


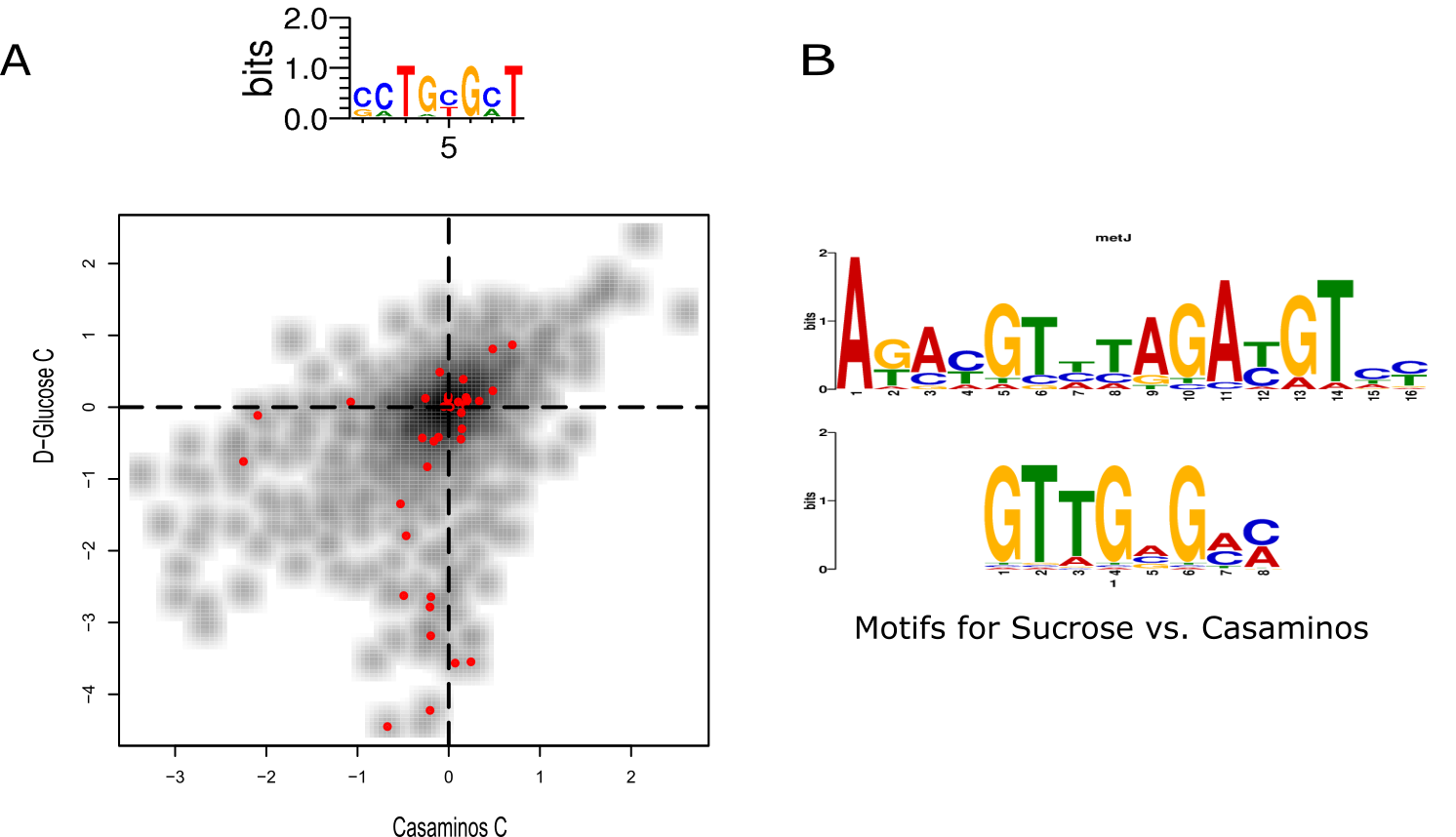


**Fig. S3.** Promoter motif analysis. **A.** The second enriched motif for D-Glucose C. vs. Casaminos C. Red points are promoters with high PSSM values with respect to the given motif. **B.** A comparison between the motif detected in Sucrose C. vs. Casaminos C (bottom) and the known metJ motif (top). MetJ logo was taken from Tomtom [24] webserver (Methods).


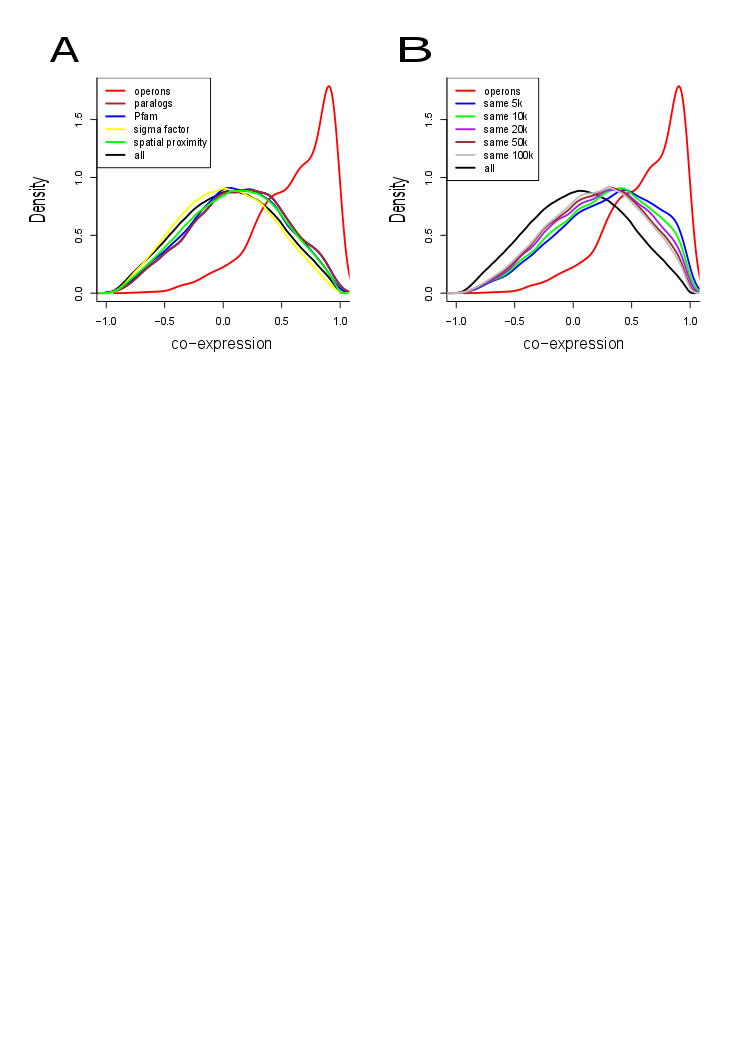
**Fig. S4.** Distributions of co-expression using different functional classes of gene pairs. Here we use density plots to represent the distributions of co-expression using different functional classes of gene pairs. Co-expression is measured by Spearman correlation. **A.** Different classes from annotation databases (Methods). **B.** Grouping the gene pairs according to genomic position bins.


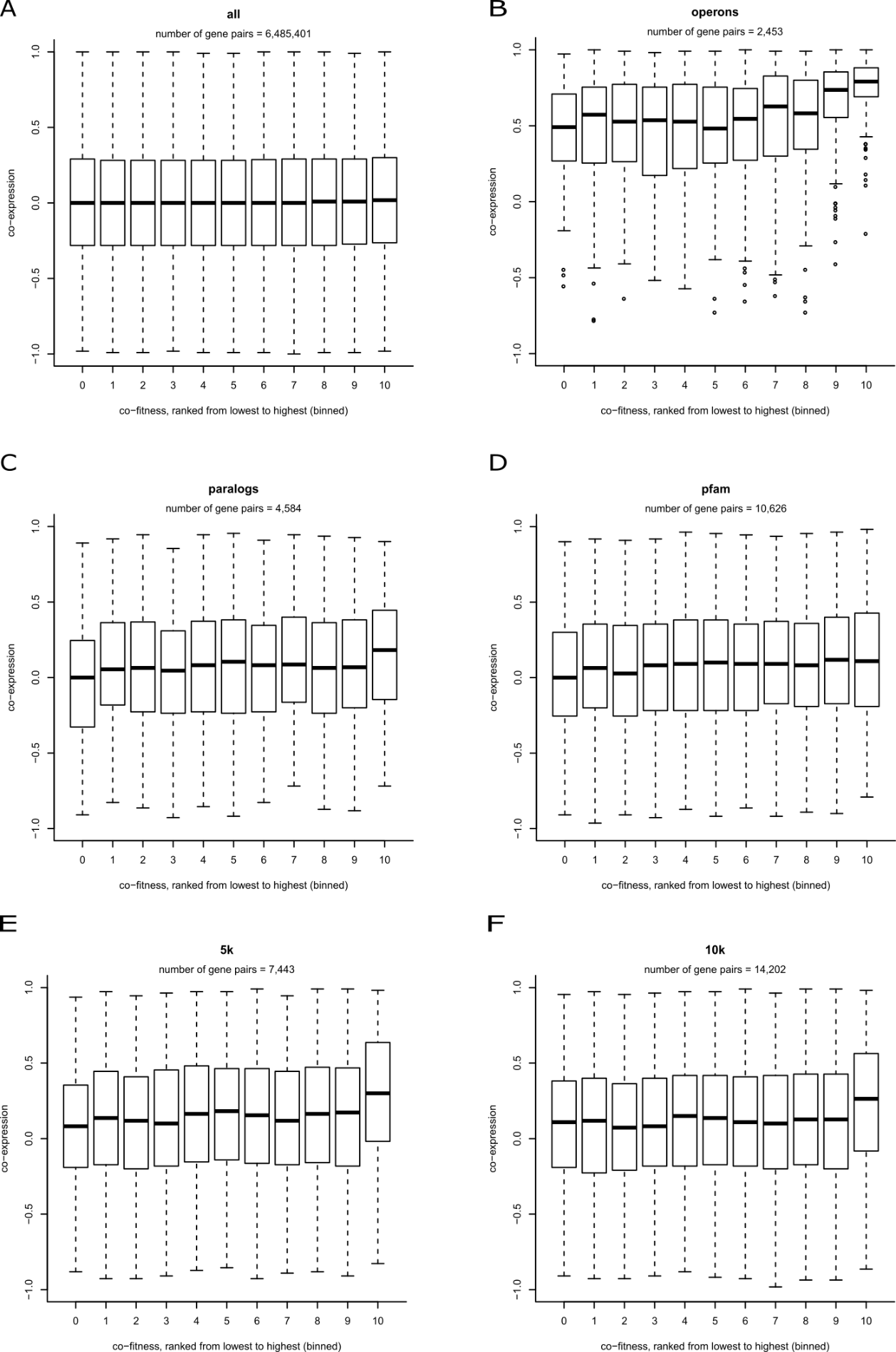


**Fig. S5.** Co-fitness and co-expression grouped using different functional classes. **A.** All gene pairs. **B.** Gene pairs within the same operon. **C.** Gene paralog pairs. **D.** Gene pairs with similar Pfam domains (at least one). **E.** Gene pairs that reside within 5kb. **F.** Gene pairs that reside within 10kb. X-axis: co-fitness, ranked from lowest to highest and binned, where each bin represents 10% of the co-fitness distribution. Y-axis: co-expression score. None of the plots represent high correlation (in all plots Spearman R between co-fitness and co-expression <0.2). Also see Spearman R values and mmHG corrected p-values in Table 1.


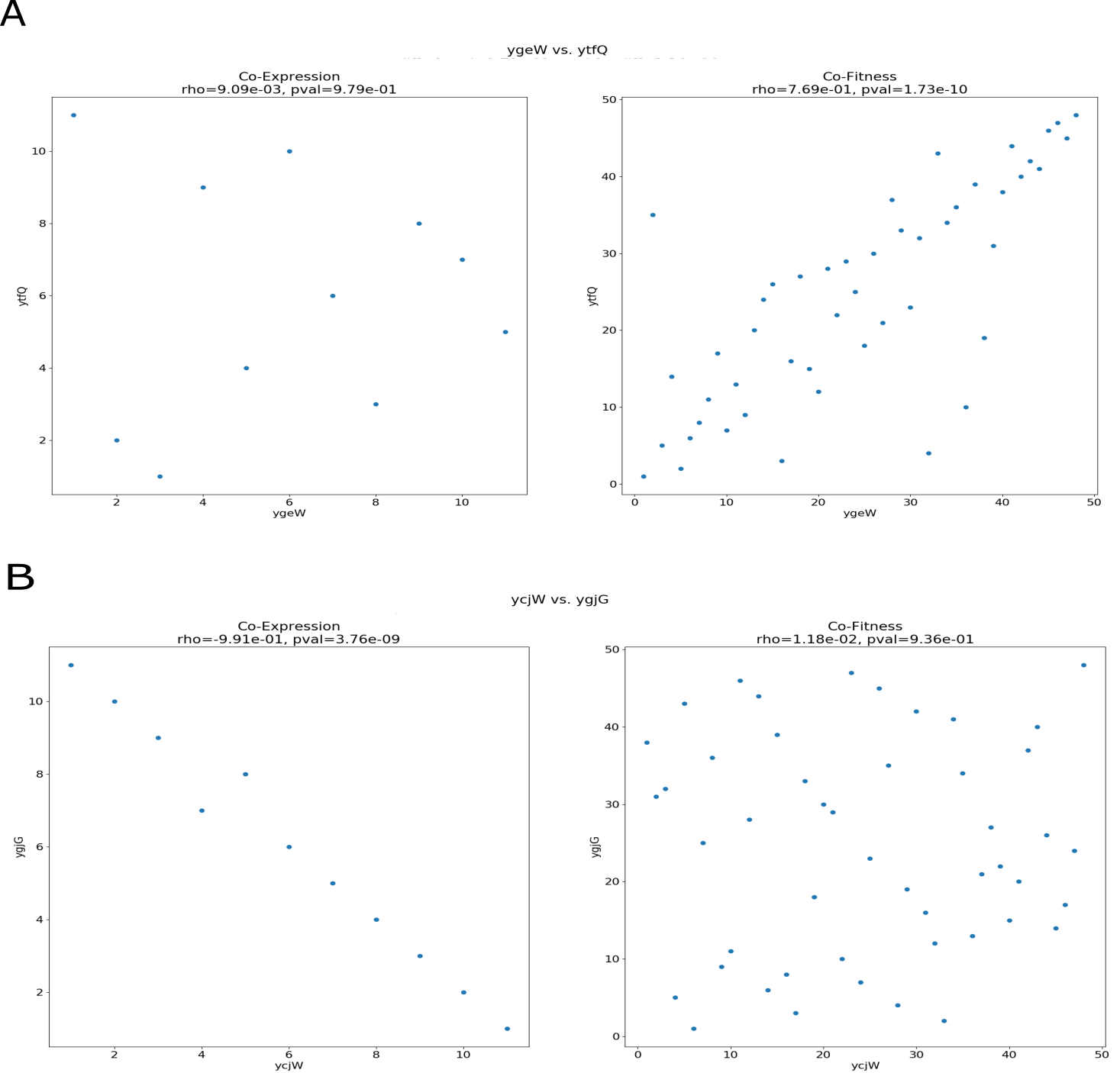


**Fig. S6.** Examples for pairs of genes with correlated fitness that are not correlated in their expression or vice versa. **A.** ygeW (b2870) fitness is correlated with ytfQ (b4227) fitness (Spearman R =0.76). However, their expression is not correlated (Spearman R =0.009). **B.** ycjW (b1320) expression is anti-correlated with ygjG (b3073) expression (Spearman R= 0.99). However, their fitness values are not correlated (Spearman R=0.011).


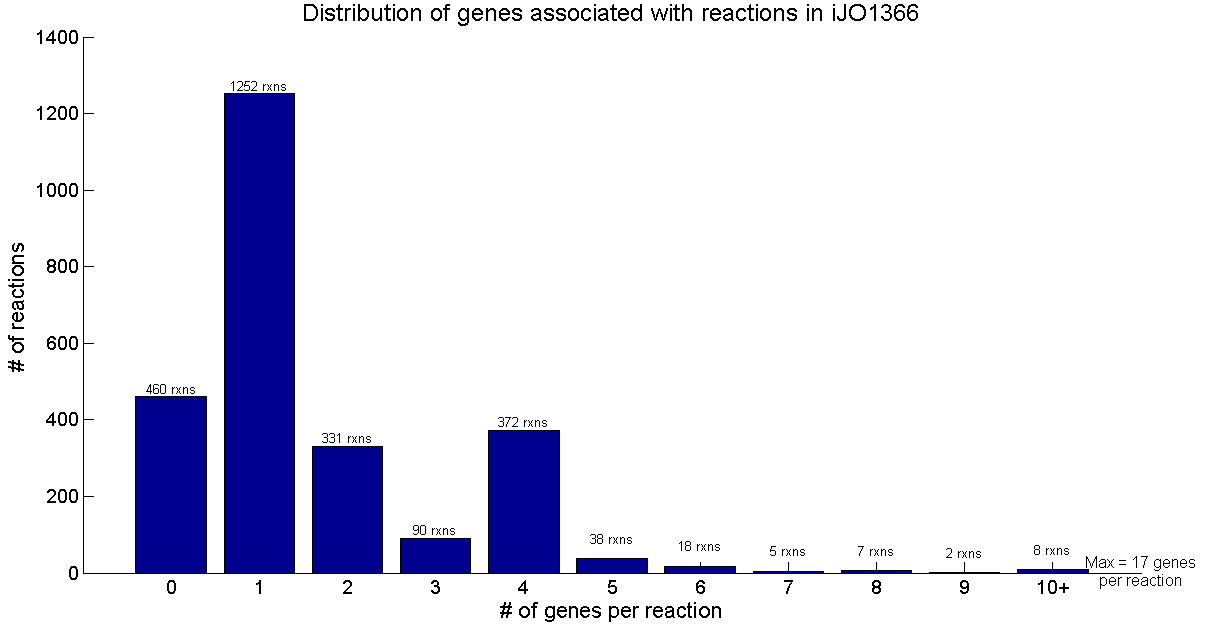


**Fig. S7.** Distribution of E. coli genes associated with reactions. This histogram depicts the number of genes assigned to each reaction in iJO1366 metabolic model of E. coli. Maximal number of genes (17) is known for Formate-hydrogen lyase reaction. Reactions without any assigned gene (total 460) belong to three major categories:

(i) Exchange reactions (330) – these reactions take metabolites to/from out-of-model space and put/get them into the extra-cellular space. These are artificial reactions and no gene can be assigned for them.

(ii) Biomass reactions (2) – these reactions describe two versions of biomass constructing reaction, as described in detail by Orth et al. These are artificial reactions and no gene can be assigned for them.

(iii) Orphan reactions (128) – these are real reactions, either transporters (58) or internal metabolic reactions (70), for which catalyzing genes are unknown. A method for predicting genes for these reactions together with the prediction results (genes associated with reactions) are among the major the contributions of the current study.


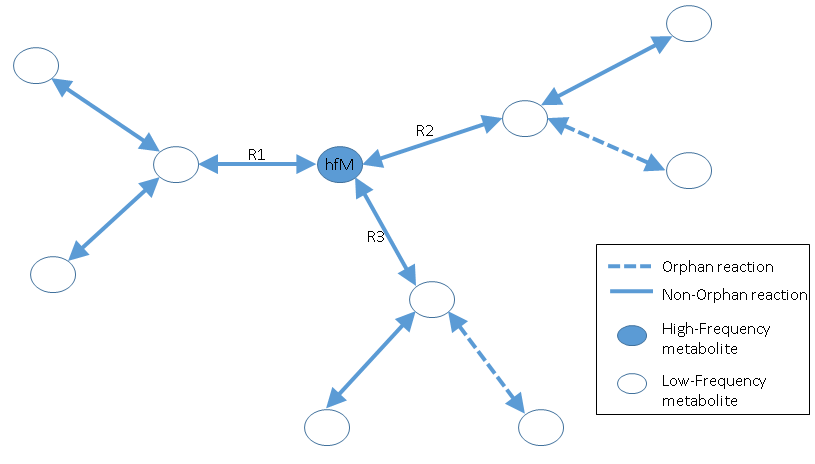
**Fig. S8.** Removing high-frequency metabolites – a greedy process. The high-frequency metabolite hfM (meaning that hfM participates in more than 11 reactions) will be removed from reaction R1, since after the removal it still has two non-orphan neighbors. However, it won’t be removed from reactions R2 and R3, since after the removal number of non-orphan neighbors will be 1 for each. The process of removing high-frequency metabolites is greedy. It starts from the metabolite with the highest frequency and iterates over its reactions in a greedy manner, starting from the one with the most neighbors. The considered metabolite is omitted from the reaction unless this reaction or any of its neighbors become inadequate, i.e. unless the number of non-orphan reaction neighbors becomes less than 2. After the reaction update is finished the process continues in the same manner to the next reaction and/or metabolite. Notice, that final definition of reaction neighbors may still depend on the order of operations in this process.


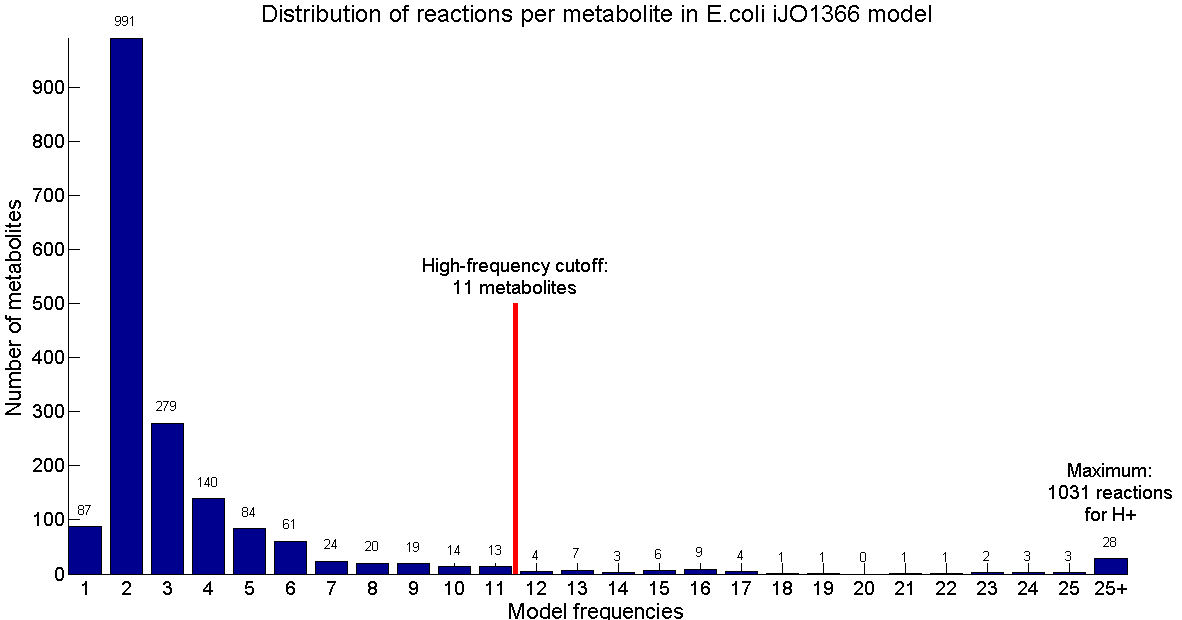
**Fig. S9.** Distribution of reactions per metabolite. This histogram depicts the distribution of metabolite appearance in the reactions of iJO1366 metabolic model of E. coli. Based on this histogram we have selected 11 as a reasonable cut-off for the definition of high-frequency metabolites.


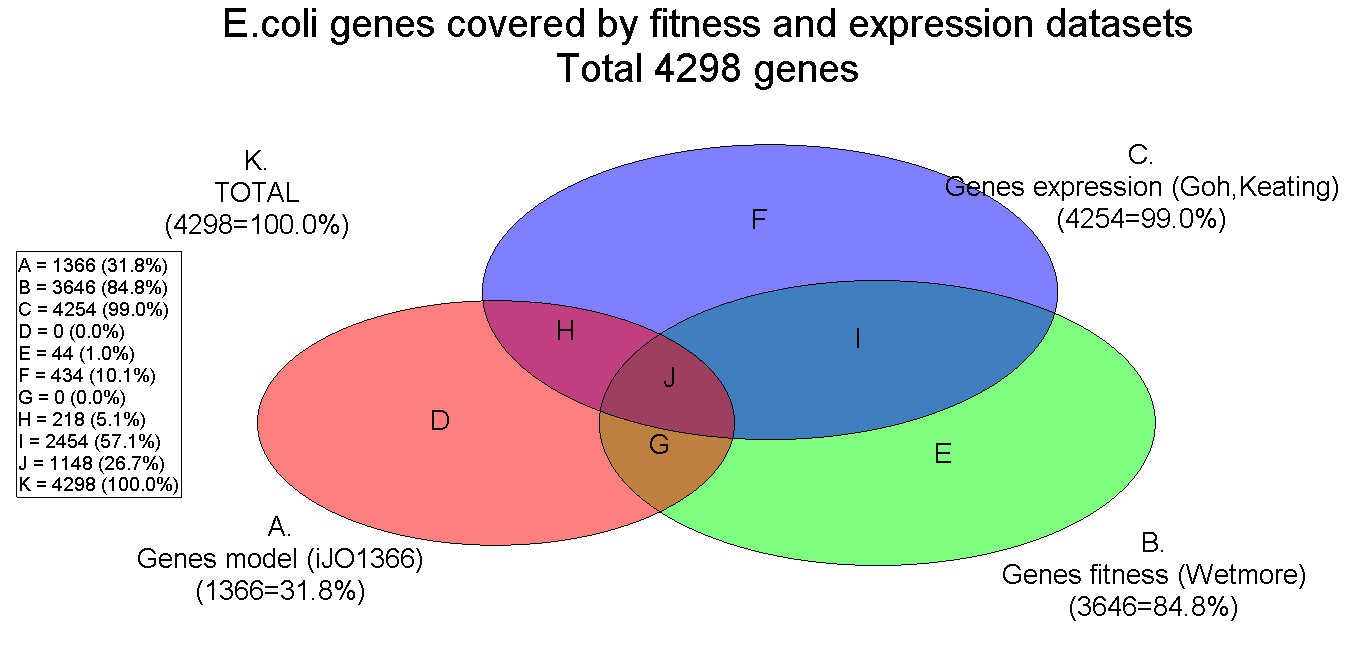
**Fig. S10.** Model genes covered by fitness and expression datasets. This diagram describes the coverage of the genes in iJO1366 model by genes existing in fitness and expression datasets.

A. 1366 genes in iJO1366 metabolic model of E. coli.

B. 3464 genes in fitness dataset from Wetmore et al.

C. 4254 genes in expression datasets from Goh et al. and from Keting et al.

D. EMPTY set of genes in iJO1366 which do not appear in expression and fitness data.

E. 44 genes in fitness data only.

F. 434 genes in expression data only. These genes were omitted from the analysis.

G. EMPTY set of genes in intersection of iJO1366 and fitness data which do not appear in expression data.

H. 218 genes in intersection of iJO1366 and expression data that do not appear in fitness data. These genes were omitted from the analysis.

I. 2454 genes from intersection of fitness and expression data that do not appear in iJO1366 model. J. 1148 genes from intersection of fitness and expression data, which are associated with at least one reaction in iJO1366 model.

K. Total number of genes (4298 genes).


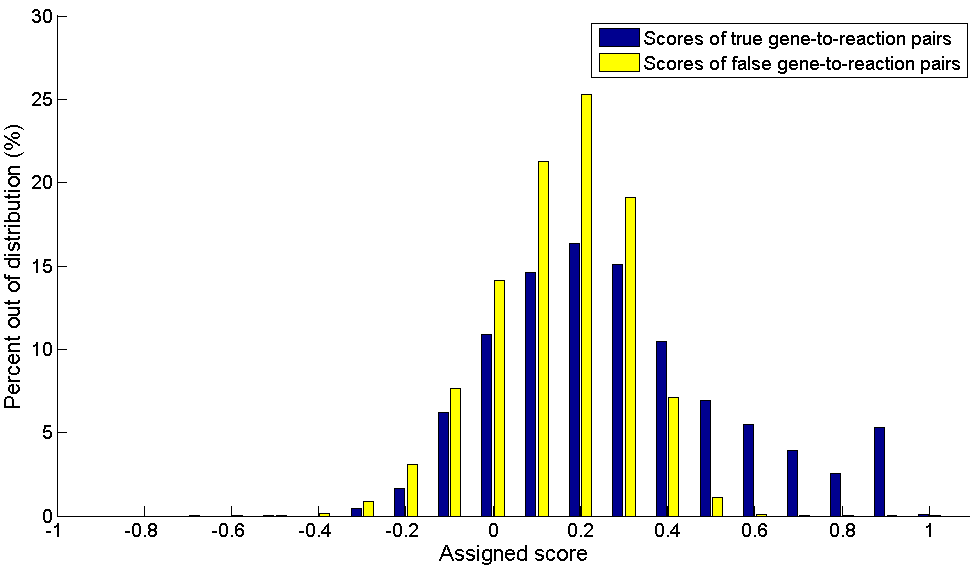
**Fig. S11.** Distribution of gene-to-reaction assignment scores. The depicted histogram describes gene-to-reaction assignment scores based on combined expression and fitness data. This histogram was created based on the validation set of non-orphan reactions, where yellow bars represent the scores of spurious gene-reaction pairs (approximately 99.96% of all pairs) and blue bars represent the scores of known gene-reaction pairs. The total number of pairs described in the histogram is ~ 6.4 M.


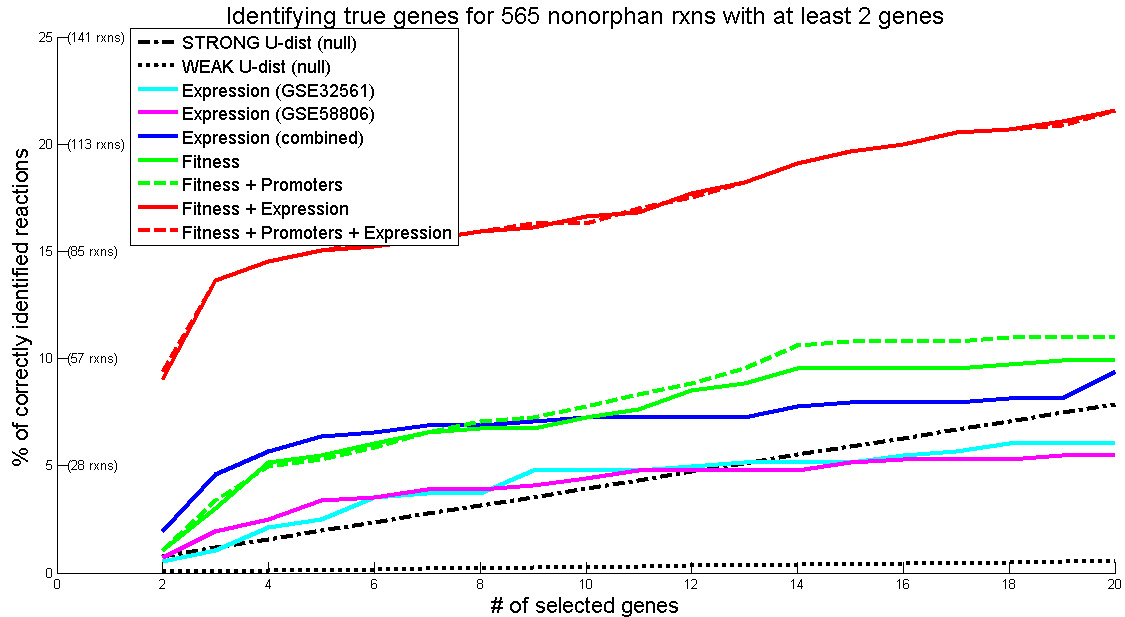
**Fig. S12.** Prediction of second reaction gene. Comparative accuracy of ALS scores predicting second reaction gene assignments for top-20 gene candidates. All the ALS scores are based on Spearman correlation to two best neighbors. Fig. S12 and Table S2 depict the accuracy of this validation predicting a of second reaction gene for 565 validation reactions with at least two genes. Notice that first reaction gene has a higher rank than a second. Here the random gene-to-reaction assignment is far more constraining and still, the performance of the assignment based both on expression and on fitness datasets has much better quality. As for the case of the prediction of the first true reaction gene, the best accuracy was obtained when combining fitness and expression datasets together and with the incorporation of promoter fitness data. This combination led to accuracy of 53 (9.4%) for correctly identifying a 2nd candidate gene (meaning that the first candidate gene is also correctly identified) and of 122 (21.6%) at the cutoff of top-20 candidates, that is an improvement of respectively 382% and 130% compared to using solely gene expression data.

Supplementary tables

Table S1. Comparative accuracy of scores predicting first reaction gene assignments. There are total 1556 adequate non-orphan reactions, i.e. those with at least one known gene and with at least two non-orphan neighbors.

| **Score** | **1st candidate** | **In top-5** | **In top-10** | **In top-20** |
| --- | --- | --- | --- | --- |
| STRONG U-dist (null) | 3.13 (0.20%) | 15.63 (1.00%) | 31.26 (2.01%) | 62.53 (4.02%) |
| Expression (GSE32561) | 106 (6.8%) | 228 (14.7%) | 262 (16.8%) | 290 (18.6%) |
| Expression (GSE58806) | 126 (8.1%) | 239 (15.4%) | 273 (17.5%) | 310 (19.9%) |
| Expression (combined) | 184 (11.8%) | 334 (21.5%) | 363 (23.3%) | 404 (26.0%) |
| Fitness | 176 (11.3%) | 413 (26.5%) | 464 (29.8%) | 503 (32.3%) |
| Fitness + Promoters | 190 (12.2%) | 425 (27.3%) | 478 (30.7%) | 513 (33.0%) |
| Fitness + Expression | 297 (19.1%) | 504 (32.4%) | 567 (36.4%) | 614 (39.5%) |
| **Fitness + Promoters + Expression** | **306 (19.7%)** | **511 (32.8%)** | **575 (37.0%)** | **617 (39.7%)** |

Table S2. Comparative accuracy of scores predicting second reaction gene assignments. There are total 565 adequate non-orphan reactions with at least two genes.

| **Score** | **2nd candidate** | **In top-5** | **In top-10** | **In top-20** |
| --- | --- | --- | --- | --- |
| STRONG U-dist (null) | 4.45 (0.79%) | 11.11 (1.97%) | 22.23 (3.93%) | 44.46 (7.87%) |
| Expression (GSE32561) | 3 (0.5%) | 14 (2.5%) | 27 (4.8%) | 34 (6.0%) |
| Expression (GSE58806) | 4 (0.7%) | 19 (3.4%) | 25 (4.4%) | 31 (5.5%) |
| Expression (combined) | 11 (1.9%) | 36 (6.4%) | 41 (7.3%) | 53 (9.4%) |
| Fitness | 6 (1.1%) | 31 (5.5%) | 41 (7.3%) | 56 (9.9%) |
| Fitness + Promoters | 6 (1.1%) | 30 (5.3%) | 44 (7.8%) | 62 (11.0%) |
| Fitness + Expression | 51 (9.0%) | 85 (15.0%) | 94 (16.6%) | 122 (21.6%) |
| **Fitness + Promoters + Expression** | **53 (9.4%)** | **85 (15.0%)** | **92 (16.3%)** | **122 (21.6%)** |
